# Ultra-Efficient PrP^Sc^ Amplification Highlights Potentialities and Pitfalls of PMCA Technology

**DOI:** 10.1371/journal.ppat.1002370

**Published:** 2011-11-17

**Authors:** Gian Mario Cosseddu, Romolo Nonno, Gabriele Vaccari, Cecilia Bucalossi, Natalia Fernandez-Borges, Michele Angelo Di Bari, Joaquin Castilla, Umberto Agrimi

**Affiliations:** 1 Department of Veterinary Public Health and Food Safety, Istituto Superiore di Sanità, Rome, Italy; 2 Department of Infectology, The SCRIPPS Research Institute, Jupiter, Florida, United States of America; 3 33458CIC bioGUNE, Parque Tecnológico de Bizkaia, Derio, Spain; 4 IKERBASQUE, Basque Foundation for Science, Bilbao, Spain; University of Edinburgh, United Kingdom

## Abstract

In order to investigate the potential of voles to reproduce *in vitro* the efficiency of prion replication previously observed *in vivo*, we seeded protein misfolding cyclic amplification (PMCA) reactions with either rodent-adapted Transmissible Spongiform Encephalopathy (TSE) strains or natural TSE isolates. Vole brain homogenates were shown to be a powerful substrate for both homologous or heterologous PMCA, sustaining the efficient amplification of prions from all the prion sources tested. However, after a few serial automated PMCA (saPMCA) rounds, we also observed the appearance of PK-resistant PrP^Sc^ in samples containing exclusively unseeded substrate (negative controls), suggesting the possible spontaneous generation of infectious prions during PMCA reactions. As we could not definitively rule out cross-contamination through *a posteriori* biochemical and biological analyses of *de novo* generated prions, we decided to replicate the experiments in a different laboratory. Under rigorous prion-free conditions, we did not observe *de novo* appearance of PrP^Sc^ in unseeded samples of M109M and I109I vole substrates, even after many consecutive rounds of saPMCA and working in different PMCA settings. Furthermore, when positive and negative samples were processed together, the appearance of spurious PrP^Sc^ in unseeded negative controls suggested that the most likely explanation for the appearance of *de novo* PrP^Sc^ was the occurrence of cross-contamination during saPMCA. Careful analysis of the PMCA process allowed us to identify critical points which are potentially responsible for contamination events. Appropriate technical improvements made it possible to overcome PMCA pitfalls, allowing PrP^Sc^ to be reliably amplified up to extremely low dilutions of infected brain homogenate without any false positive results even after many consecutive rounds. Our findings underline the potential drawback of ultrasensitive *in vitro* prion replication and warn on cautious interpretation when assessing the spontaneous appearance of prions *in vitro*.

## Author Summary

In an attempt to transpose to an *in vitro* system the particular sensitivity of the vole model to human and animal Transmissible Spongiform Encephalopathies (TSEs), we first explored the suitability of vole brain homogenate as a substrate for PMCA. As well as observing the highly efficient replication of a variety of prion sources, we also found preliminary evidence of *de novo* prion generation in unseeded reactions. Careful analysis of the PMCA procedure, undertaken further to investigate these findings, showed, however, that they were the result of cross-contamination with seeded samples. We next identified and investigated the critical points of this procedure that are potentially responsible for cross-contamination. Our results suggest that *in vitro* systems for prion amplification could be more prone to cross-contamination than previously thought, particularly when harsh procedures, such as sonication, are involved. Experimental conditions able to reproduce spontaneous prion formation in a simple and easily reproducible *in vitro* system would be of crucial interest for understanding TSEs and other important neurodegenerative disorders. Given that *in vitro* methods are increasingly used in this field, our results emphasise the possible drawbacks of such approaches and call for the use of rigorously controlled conditions and cautious interpretation of data.

## Introduction

Transmissible Spongiform Encephalopathies (TSEs) are progressive and fatal neurodegenerative disorders that include scrapie of sheep, bovine spongiform encephalopathy (BSE) of cattle and Creutzfeldt-Jakob disease (CJD) of humans [1]. The nature of the causal agent of TSEs has long been a matter of intense scientific debate. The prion hypothesis postulates that the causal agent, the prion, consists only of proteins without nucleic acid genome [1]. Alternative hypotheses postulate the presence of a small nucleic acids genome [2], although evidences for this are still lacking. The virino hypothesis proposes that the causal agent is an informational hybrid between the agent genome and host conformationally altered PrP [3]. Recently, new evidences were brought in support of the prion hypothesis, although a fundamental role of non proteinaceous cofactors could not be definitively excluded [4], [5], [6], [7], [8]. The accumulation in the central nervous system of a post-translationally altered isoform (PrP^Sc^) of the cellular prion protein (PrP^C^) is the key event in TSE pathogenesis [1], Nevertheless the relationships between PrP^Sc^ and infectivity are not definitively clear and evidences for high titers of TSE infectivity associated with extremely low levels of PrP^Sc^ have been reported [9]. The modification of PrP^C^ involves mostly unknown conformational changes during which an increase in the amount of β-sheet of the normal protein and a decrease in its α-helical content is observed [10], [11]. According to the prion theory, PrP^Sc^ thus acquires, via a template-based mechanism, the ability to trigger the conversion of PrP^C^ into new PrP^Sc^. The process proceeds thereafter in an autocatalytic manner, leading PrP^Sc^ aggregates to grow by including new PrP^C^ monomers [1], [12].

The severe outbreak of BSE, first detected in the UK in 1986, and the announcement in 1996 that the BSE agent was responsible for a newly recognised form of TSE in humans, named variant CJD, created enormous concern among European consumers and prompted health authorities to promote the development of reliable diagnostic tools for BSE [13], [14], [15]. Recently, four cases of transfusion-related transmission of vCJD [16] further strengthened the need for reliable preclinical and *in vivo* screening tests for TSEs. The protein misfolding cyclic amplification (PMCA) technology developed in 2001 by Claudio Soto's group [17], seems one of the most promising approaches. PMCA is used to amplify minute amounts of PrP^Sc^ existing in the test material to levels which can be readily detected using conventional assays such as Western blotting. The reaction is initiated by diluting TSE-infected material in normal brain homogenate, with the former providing PrP^Sc^ seeds and the latter providing PrP^C^ and other potential cofactors for PrP^Sc^ amplification. The product is diluted in additional normal brain homogenates for subsequent serial amplification cycles that allow theoretically indefinite PrP^Sc^ amplification [18]. PMCA has usefully demonstrated that prion infectivity can be replicated *in vitro* [18] and that *in vitro*-generated prions maintain apparently unaltered strain properties [19], [20]. Moreover, PMCA-induced replication of prion seems to reproduce *in vitro* several aspects of the *in vivo* replication and is valuable for investigating the molecular requirements for PrP trans-conformation [4] and to mimic the intra- and inter-species replication and adaptation of prions [21]. Finally, PMCA has demonstrated its ability to achieve ultrasensitive detection of PrP^Sc^ in tissues and body fluids of TSE-affected rodent models [22], [23], [24].

In spite of its usefulness for investigating basic aspects of TSEs, very few data have been published proving the robustness of PMCA as a diagnostic tool for natural TSEs and the appearance of spurious results has been highlighted in some papers [25], [26]. PMCA is hampered by technical difficulties that make improvements necessary in terms of both practicability and control. For diagnostic purposes the sensitivity and specificity of PMCA need to be close to 100%. However, the appearance of protease-resistant bands identical to PrP^Sc^, in PrP^Sc^ inoculum-free samples reported by Soto's group when more than 10 rounds of standard PMCA were performed [25] and the recent papers claiming *de novo* generation of infectious prions in unseeded PMCA reactions [4], [27] raise fundamental questions about the diagnostic reliability of PMCA. If the latter findings will be corroborated by replication in different laboratories, they would assume critical importance in the interpretation of PMCA results and in its possible clinical-diagnostic use.

A practical limitation of PMCA is the reported need to use a substrate that is compatible with the target to be amplified. In an attempt to transpose to an *in vitro* system the plasticity shown by the bank vole model in the transmission of a variety of human and animal TSEs [28], [29], [30], we explored the suitability of vole brain homogenate as a substrate for PMCA. Together with the observation of a highly efficient replication of a variety of prion sources from different species, the earliest experiments produced apparently conflicting results: after serial PMCA rounds using healthy vole brain homogenates without infectious seed, the appearance of PK-resistant PrP^Sc^ was repeatedly observed. This product proved to be very infectious when inoculated into voles, thus suggesting that prions can be generated *de novo* from healthy brains. In the present study we illustrate, as supplementary on-line material, our early evidence of the, *de novo* generation of prions and describe in detail the procedures we performed to explore further and validate those results. When working in rigorously prion-free conditions, we found no evidence of *de novo* generation of prions. Through careful analysis of the PMCA process we identified the critical points of this procedure that are potentially responsible for contamination events and false positive results when ultrasensitive detection is desired. Here we show that technical improvements can be adopted to overcome the drawbacks of PMCA, and that PrP^Sc^ can be reliably amplified from very high dilutions of infected brain homogenate in a single PMCA round.

## Results

### 1. Vole PMCA: early results

**Figure 1:**
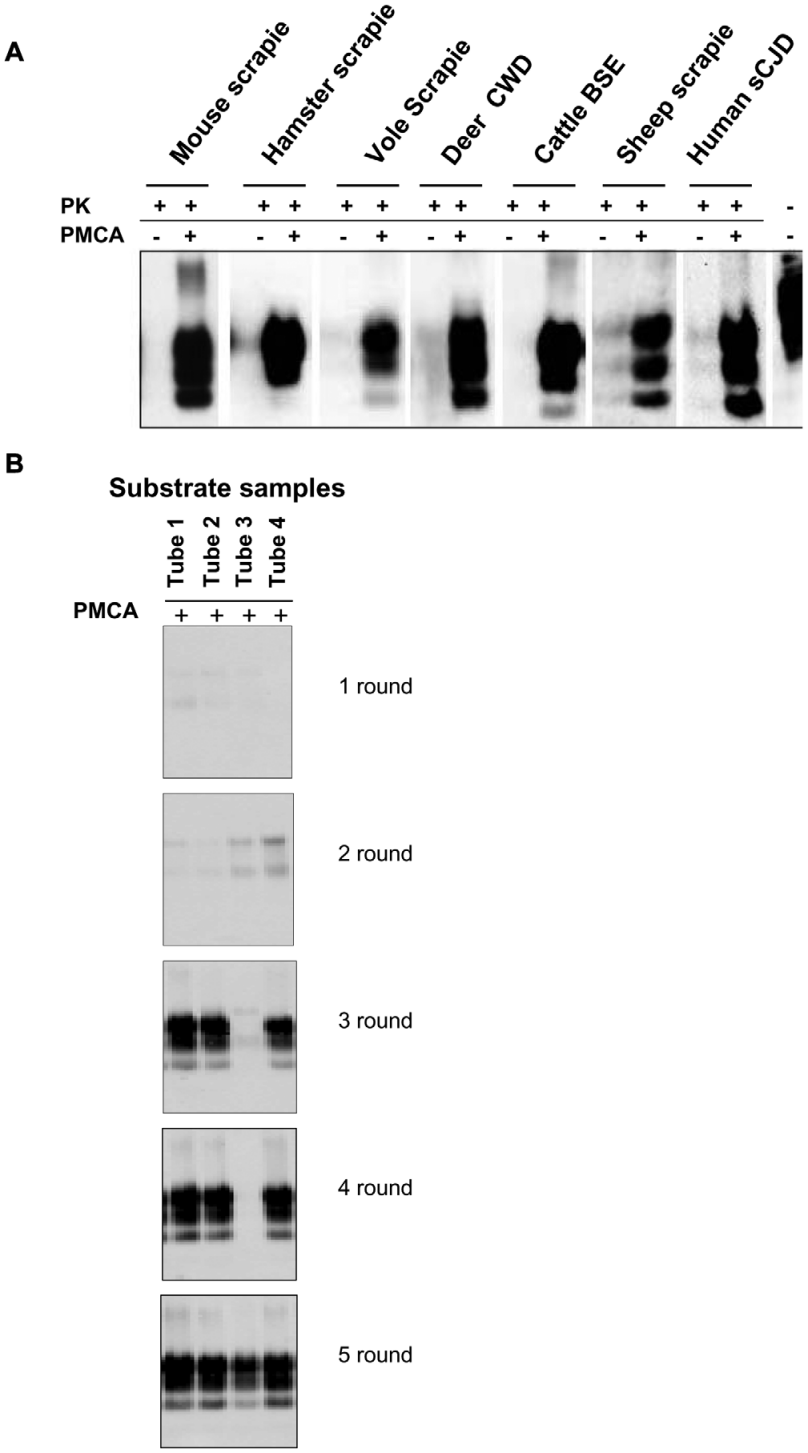
Vole PMCA: early results. **A**) The efficiency of vole substrate to support the amplification of PrP^Sc^ from different prion sources: 10% infected brain homogenate from hamster, mouse and bank vole with experimental scrapie, deer with natural CWD, cattle with natural BSE, sheep with scrapie and humans with type 1 sCJD were diluted 1∶200 in substrate from voles carrying M109M PrP genotype and amplified for 80 consecutive sonication/amplification cycles. After amplification PMCA material was PK-digested and separated by SDS-PAGE; Western blot was probed with anti-PrP 6D11 primary antibody. **B**) The putative *de novo* generation of PrP^Sc^ from healthy brain homogenates: samples of vole substrate carrying the M109M PrP genotype, (tubes 1 to 4) without any positive inoculum were amplified for 5 consecutive rounds of saPMCA (24 sonication/incubation cycles per round). After the third round the presence of PrP^Sc^ was observed in 3 out of 4 samples. After the 5 consecutive rounds all samples were positive. After amplification, PMCA material was PK digested and separated by SDS-PAGE; Western blot was probed with anti-PrP D18 primary antibody.

We previously reported the susceptibility of bank voles to different prion species, including human, rodent, cattle, sheep and elk TSEs [28, 29, 30 and manuscript in preparation]. Voles have a polymorphism at codon 109 of PrP, coding for either methionine or isoleucine, and this amino acid variation influences the susceptibility of voles to different prion strains [31 and Agrimi et al., unpublished data]. In order to investigate the potential of voles to reproduce *in vitro* the efficiency of prion replication previously observed *in vivo*, we seeded PMCA reactions either with rodent-adapted prion strains, including mouse- and vole-adapted scrapie and BSE strains and hamster-adapted scrapie 263K, and with natural isolates of sheep scrapie, chronic wasting disease of elk, cattle BSE, MM1 sCJD, to a final seed homogenate/vole substrate dilution of 1/200. Vole brain homogenates were indeed a powerful substrate for PMCA, supporting the efficient amplification of PrP^Sc^ from all the prion sources tested, either derived from voles or other species, after a single PMCA round of 80 amplification cycles (Figure 1A). However, after a few serial automated PMCA (saPMCA) rounds, we also observed the appearance of PK-resistant PrP^Sc^ in samples containing exclusively unseeded substrate (negative controls) (Figure 1B). This finding suggested the possible occurrence of spontaneous generation of PrP^Sc^ during PMCA reactions (see Supplemental Text S1).

Additional PMCA experiments performed in similar conditions confirmed the putatively *de novo* appearance of PrP^Sc^ in unseeded brain homogenates from M109M and I109I voles (Table S1). In order to exclude the possibility that animals from the facility of Istituto Superiore di Sanità (ISS) were contaminated, PMCA was also carried out using substrates from wild voles, trapped in the countryside, with similar results (see footnotes in Table S1). Overall, the above experiments showed that: i) the supposedly spontaneous appearance of PrP^Sc^ was a stochastic event, with each individual sample acting independently (Table S1); ii) two different PrP^Sc^ types could be generated during unseeded PMCA reactions in either M109M and I109I vole substrates (Figure S1); and iii) there was no obvious correlation between the age of the voles used for preparing PMCA substrates and the relative ability to generate supposedly *de novo* PrP^Sc^ (Table S1). A detailed WB analysis of PrP^Sc^ types obtained from unseeded PMCA reactions in M109M and I109I vole substrates showed that the supposedly *de novo* generated PrP^Sc^ types could not be unequivocally distinguished from some of the vole-passaged prion strains used in our laboratory (Figures S2, S3).

Bioassay of brain homogenates after several consecutive rounds of saPMCA showed that PrP^Sc^ from either seeded and unseeded positive PMCA reactions were infectious after intracerebral inoculation of voles (Tables S2, S3 and Text S2). Biological strain typing in M109M voles showed that the two different *de novo* PrP^Sc^ types induced two easily distinguishable prion strains, based on survival times (Table S2), WB analysis of PrP^Sc^ (Figure S4) and brain regional distribution of PrP^Sc^ deposition and spongiform degeneration (Figures S5, S6 and Text S2). However, these two putatively spontaneous prion strains could not be unequivocally discriminated from other PMCA-passaged prion strains used in our experiments (Table S3 and Figure S6).

### 2. No evidence of *de novo* generation

Considering the importance of demonstrating the spontaneous generation of prions in experimental conditions and our inability to unequivocally exclude cross-contamination by *a posteriori* biochemical and biological analyses of *de novo* generated prions, we decided to confirm our preliminary results by replicating the experiments in a different laboratory. A new series of experiments was therefore performed in a prion-free laboratory at ISS by the same person who obtained the preliminary results at the SCRIPPS Institute (GMC). Importantly, during these experiments no positive controls were used, as they had been in the previous experiments; in this way there was no danger of any cross-contamination event and the laboratory was maintained prion-free.

Eleven independent saPMCA experiments were carried out using exclusively unseeded substrate samples, which were processed for 6–13 consecutive rounds (Table 1). During these experiments several factors were tested which could potentially influence the spontaneous generation of prions. These were: genetic vole PRNP genotype (M109M or I109I), individual age of the animals, power of sonication, duration of sonication or incubation and the number of cycles in each round. All the conditions tested are reported in detail in Table 1. All saPMCA samples from each round of the above experiments were analysed by Western blot. No samples showed a signal compatible with genuine PrP^Sc^.

**Table 1:** Unseeded saPMCA experiments carried out in prion free laboratory.

ID	PMCA conditions						
	Tubes per round	Individual substrates (PrP genotype and age class)		Sonic/Incub. cycles	Power of sonic. Watts (potency)	Serial rounds	hours/cycles per round
		*Met109Met*	*Ile109Ile*				
1	30	3(a)	2(a)	20 sec./30 min	200 (7)	9	24/48
2	12	1(b)	1(b)	20 sec./30 min	200 (7)	10	24/48
3	12	1(c)	1(c)	20 sec./30 min	200 (7)	13	24/48
4	12	1(d)	1(d)	20 sec./30 min	200 (7)	12	48/96
5	16	1(a), 1(b)	1(a), 1(b)	20 sec./30 min	200 (7)	9	48/96
6	16	1(b), 1(c)	1(b), 1(c)	20 sec./30 min	290 (10)	9	12/24
7	30	3(b)	2(b)	20 sec./30 min	260 (9)	10	12/24
8	75	3(a), 3(b)	3(a), 3(b)	20 sec./30 min	240 (8)	9	8/16
9	70	3(a), 4(b), 3(c)	1(a), 1(b), 2(c)	20 sec./30 min	200 (7)	8	48/96
10	72	3(a), 3(b), 3(c), 3(d)	0	20 sec./30 min	200 (7)	6	24/24
11	20	3(b), 2(c)	0	2 sec./30 Ssec	200 (7)	6	24/48

Footnote to Table 1. Eleven independent experiments were carried out in the prion free laboratory using 4 to 6 replicates of unseeded substrate, in absence of any positive inoculum. This table provides details on each experiment reporting the number substrates samples, the PrP genotype and the age of the individual voles. Animals belong to four classes of age: a = 4–8 weeks old; b = 2–4 months old; c = 5–12 months old; d =  older than 1 year. The settings of saPMCA (the length of incubation and sonication cycles, the power of sonication, number of consecutive rounds and the length of rounds) are also given.

**Figure 2:**
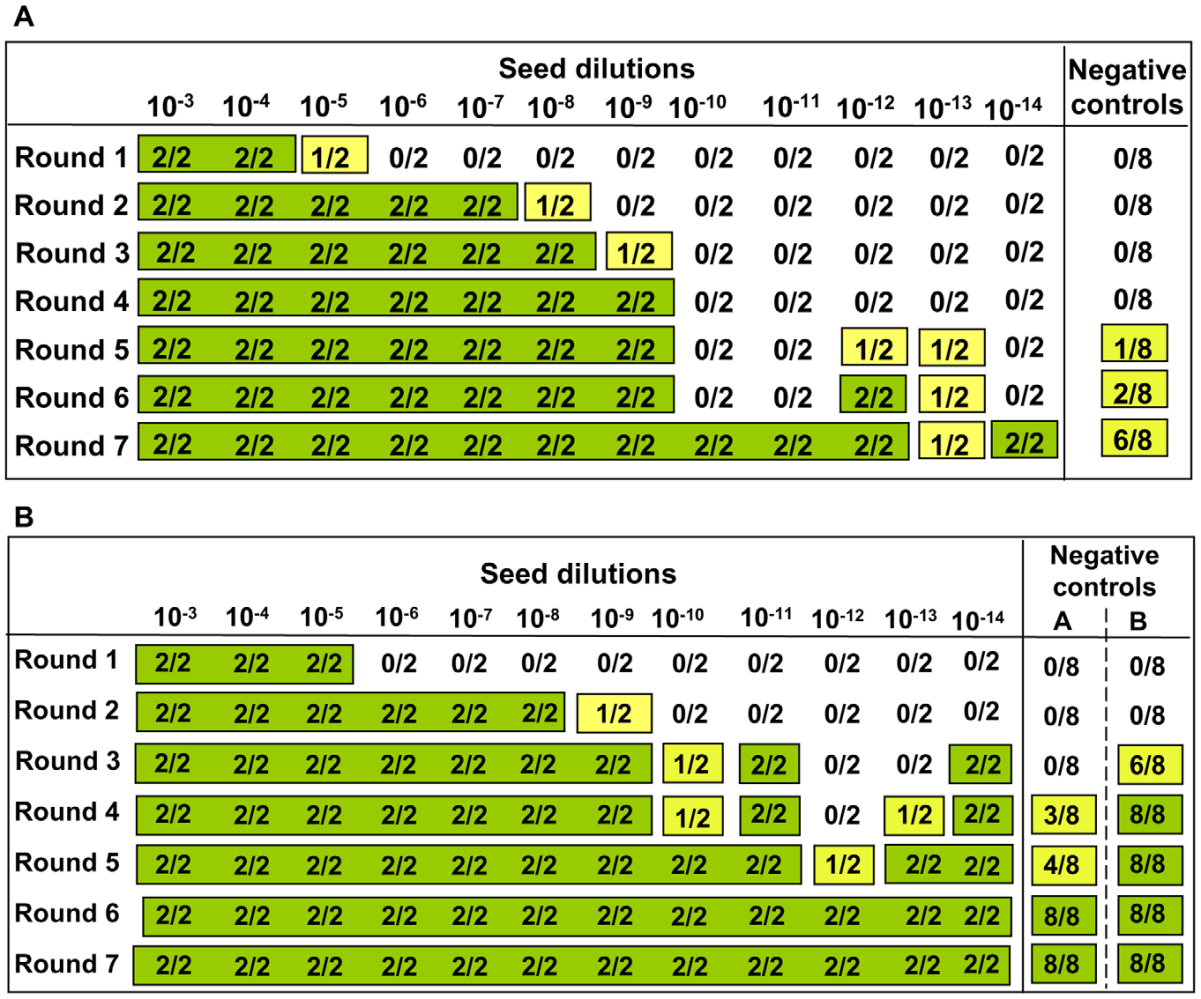
Sensitivity of detection of saPMCA using vole substrate and v586 inoculum. For each round and seed dilution is reported the number of positive samples over the number of replicates tested. Green boxes indicate that all replicates were positive; yellow boxes indicate that less than 100% of replicates were positive, while boxes are white when no positive replicates occurred. **A**) Logarithmic dilutions of the seed were prepared in duplicate from 10^−3^ to 10^−14^ using vole M109M substrate. Eight negative controls were included. Samples were amplified for 7 consecutive rounds of 24 hours. All the samples were analysed by Western blot. Results showed that after the first round, amplifications were observed in dilutions 10^−3^, 10^−4^ and in one of two duplicates of 10^−5^. After the second round, positive PrP^Sc^ signals were detected also in 10^−5^, 10^−6^, and 10^−7^ dilutions and in one of the 10^−8^ duplicates. After three consecutive rounds, 10^−9^ reached positive amplification. Lower dilutions were amplified between the 5^th^ and 7^th^ round but, at the same time, also negative controls became positive. **B**) logarithmic dilutions of v586 in duplicate from 10^−3^ to 10^−14^ were amplified for 7 consecutive 24-hour rounds using vole M109M substrate. Two groups of 8 unseeded negative controls were included.(groups A and B). All the seeded and unseeded samples were amplified together, but passages of control group A were carried out in a prion-free environment (new laminar flow hood and pipette), while samples from control group B were processed together with the seeded tubes. Results showed similar sensitivity compared to previous experiments, with amplification of 10^−5^ dilution after the first round and up to 10^−9^ after the second round. After the third round the 10^−10^, 10^−11^ and 10^−14^ dilutions along with six out of eight negative samples from control group B become positive. One round later, three out of eight control group A samples also turned positive.

**Figure 3:**
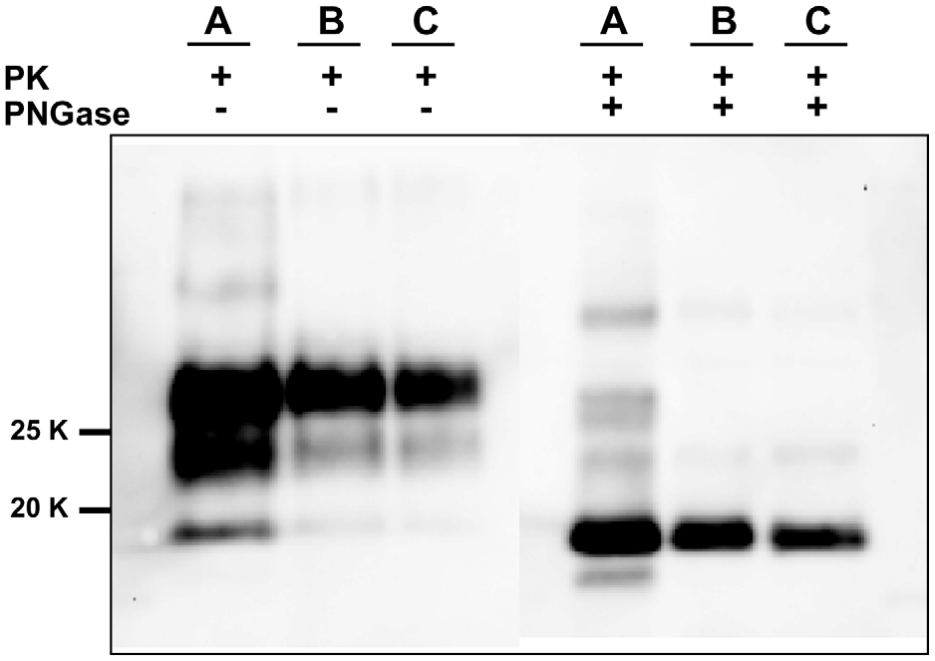
Comparison between PrP^Sc^ from seeded and unseeded PMCA reactions. Characterization of PrP^Sc^ from positive samples of the experiment described in Figure 2A by Western blot, stained with SAF84 primary antibody. The figure shows that samples A (PrP^Sc^ from 10% brain homogenate used as positive inoculum), B (10^−5^ dilution of the inoculum after a single 24-hour round of PMCA) and C (PrP^Sc^ from an unseeded negative control which was positive after 6 consecutive rounds of saPMCA) share the same apparent molecular weight before or after deglycosylation with PNGase.

As no seed (positive control) was included in the experiments, the efficiency of all substrates used in previous experiments and the efficiency of the different PMCA settings used (Table 1) were further tested. For these experiments, the same substrates and PMCA settings described in Table 1 were used in PMCA experiments seeded with v586 prion strain (see Methods). All substrates and all PMCA conditions successfully amplified the seed to the 10^−3^/10^−5^ dilution in a single PMCA round of 24 hours (Figure S7 and data not shown). However it can be hypothesised that the *de novo* appearance of prions requires a high amplification efficiency. We therefore evaluated the sensitivity of our saPMCA by amplifying serial dilutions (from 10^−2^ to 10^−14^) of the v586 strain in healthy vole brain homogenates in consecutive rounds of 24 hours in standard conditions. The results are shown schematically in Figure 2A. The sensitivity of detection was very high, the 10^−9^ dilution being positive after 3 rounds. However, after the fifth round both very high dilutions of the seed and unseeded tubes became positive. This experiment was repeated with similar results (not shown). The molecular profile of PrP^Sc^ recovered from seeded and unseeded reactions revealed a molecular pattern identical to the v586 strain used as inoculum (Figure 3). Taken together, and given that the only difference in comparison with previous experiments reported in Table 1 was the presence of a positive seed, these data strongly suggest that the *de novo* appearance of PrP^Sc^ in unseeded tubes could have been due to cross-contamination.

Given that prion-infected samples had been introduced, the laboratory was no longer considered prion-free. Because of the contrasting results obtained at SCRIPPS and at ISS, a further and final attempt to test the possibility of *de novo* generation of prions was made. Skilled personnel (NF) from the SCRIPPS Research Institute joined the local staff at ISS (GMC) to perform a new series of saPMCA experiments under prion-free conditions. The experiment was carried out in a bacteriology laboratory that had never been used for prions. A new sonicator (Misonix S4000), gravity oven and thermal bath were bought, along with pipettes, Potter type glass homogenisers, consumables and reagents. Seventy-two unseeded bank vole substrate samples (derived from 11 M109M and 4 I109I voles) were submitted to saPMCA for 10 consecutive rounds of 24 hours, using standard settings. The experiment was monitored by analysing, by Western blot, all the 720 PMCA products. As with previous experiments with unseeded samples (Table 1), no evidence of PrP^Sc^ generation was observed in any of the samples (data not shown). Immediately after the completion of the experiments, the sonicator was moved to our prion laboratory in order to test the efficiency of the substrates and the proper functioning of the sonicator. Amplification of dilutions from 10^−3^ to 10^−6^ was attained with all the substrates after 1 round of 24 hours (data not shown), demonstrating the efficiency of our PMCA conditions and the consistency of the negative results previously obtained.

Overall the above results show that the supposedly *de novo* appearance of PrP^Sc^ in unseeded samples observed in our previous experiments was not replicated when working in an absolutely prion-free laboratory (i.e. without using positive controls), even after many consecutive rounds of saPMCA and in different experimental conditions. By contrast the appearance of spurious PrP^Sc^ in unseeded negative controls when positive and negative samples were processed together (Figure 2A), suggests that the most likely explanation for the appearance of *de novo* PrP^Sc^ previously observed is the occurrence of cross-contamination during saPMCA.

### 3. Focusing on contamination

The occurrence of cross-contamination during saPMCA raises serious doubts about the advisability of using PMCA for the detection of prions, particularly when ultrasensitive amplification is desired. We therefore focused on identifying the critical points where cross-contamination could occur and the best conditions for sensitive and specific amplification. In principle, two critical points at which contamination could have occurred were identified: 1) in the sonicator, during repeated cycles of sonication/incubation; 2) during manipulation of samples between PMCA rounds. To assess which of these two steps was responsible for cross contamination, serial dilutions of the v586 prion strain and two distinct groups of unseeded negative controls (A and B) were amplified for 7 consecutive rounds (Figure 2B). All tubes were amplified and processed together, except that the tubes of control group A were handled separately after each round in order to avoid cross-contamination during manipulation of samples. Passages of control group A were carried out in a new laminar flow hood using a new pipette, while passages of control group B were carried out together with seeded samples. The results were comparable to those obtained in previous experiments (compare Figures 2A and 2B), with control tubes of both group A and B showing positive signals after 4 and 3 PMCA rounds, respectively. A single PrP^Sc^ molecular signature, identical to the v586 inoculum, was observed in all positive samples (data not shown). As the tubes used for control group A were never exposed to possible sources of contamination outside the sonicator, these data imply that cross-contamination occurs in the sonicator during the amplification cycles, although they do not allow us to exclude that tubes may also become contaminated during passages. To check if the sonicator was able to retain PrP^Sc^ molecules and to release them later as a source of contamination in subsequent experiments, we then serially amplified unseeded samples in the sonicator just used for the previous experiments. Passages were carried out under the flow hood and with the pipette used for previous seeded experiments. Following 10 consecutive rounds under standard PMCA conditions, no positive sample was obtained (data not shown), showing that the sonicator, the flow hood and the pipette previously used for seeded PMCA reactions are not a strong source of contamination *per se*.

Overall the above results indicate that the simultaneous manipulation and processing of seeded and un-seeded samples is critical and may easily give rise to cross-contamination events, with the most likely critical factor being the simultaneous presence of seeded and unseeded tubes in the sonicator during the amplification cycles. Moreover, they also indicate that indirect contamination of the environment (benches, laminar flow hood) and instruments (pipettes, sonicator, thermal bath) from previous seeded experiments is not critical, provided that general measures to limit it are taken.

### 4. Pursuing cross-contamination control

During PMCA, the sonication of substrates produces mechanical shearing inside the reaction tube that might force opening of the vial, thus exposing unseeded samples to cross-contamination. It is also possible that small prion particles could penetrates through microscopic cavities between the tube wall and cap contaminating water in the horn and non-seeded samples. The 0.2 mL PCR tubes used for PMCA do not lock and are intended to be used in a thermal cycler. In effect, the caps of the tubes in the thermal cycler are normally tightened by the pressure of a heated lid. In order to improve the tightness of the 0.2 mL reaction tubes, we first sealed the tubes with a small Parafilm M strip positioned on the external junction between the tube and its cap, and then developed a “sealer” with a moving arm equipped with a sonicator probe. When the probe was applied to the cap of the vial, it was fastened to the tube by the heat generated by short sonication pulses. Both procedures certainly improved the tightness of the tubes, although when serial PMCA was performed they proved inadequate to prevent cross-contamination completely (data not shown).

Subsequently we evaluated new vials bought on the market. We selected Multiply-Safecup Biosphere, handy 0,5 mL tubes with screw cap and 100 µl volume limitation that works as a double closure system (Figure S8). The ability of the new 0,5 mL screw-cap vials to support high amplification levels was evaluated by amplifying serial dilutions of v586 inoculum. No difference was observed when we compared the efficiency of the 0,2 ml vial and the 0,5 mL screw-cap tubes (data not shown). Importantly, the screw-cap tubes were able completely to prevent cross-contamination, even after many consecutive rounds (see below).

### 5. Achieving ultrasensitive amplification and control of cross-contamination during saPMCA

**Figure 4:**
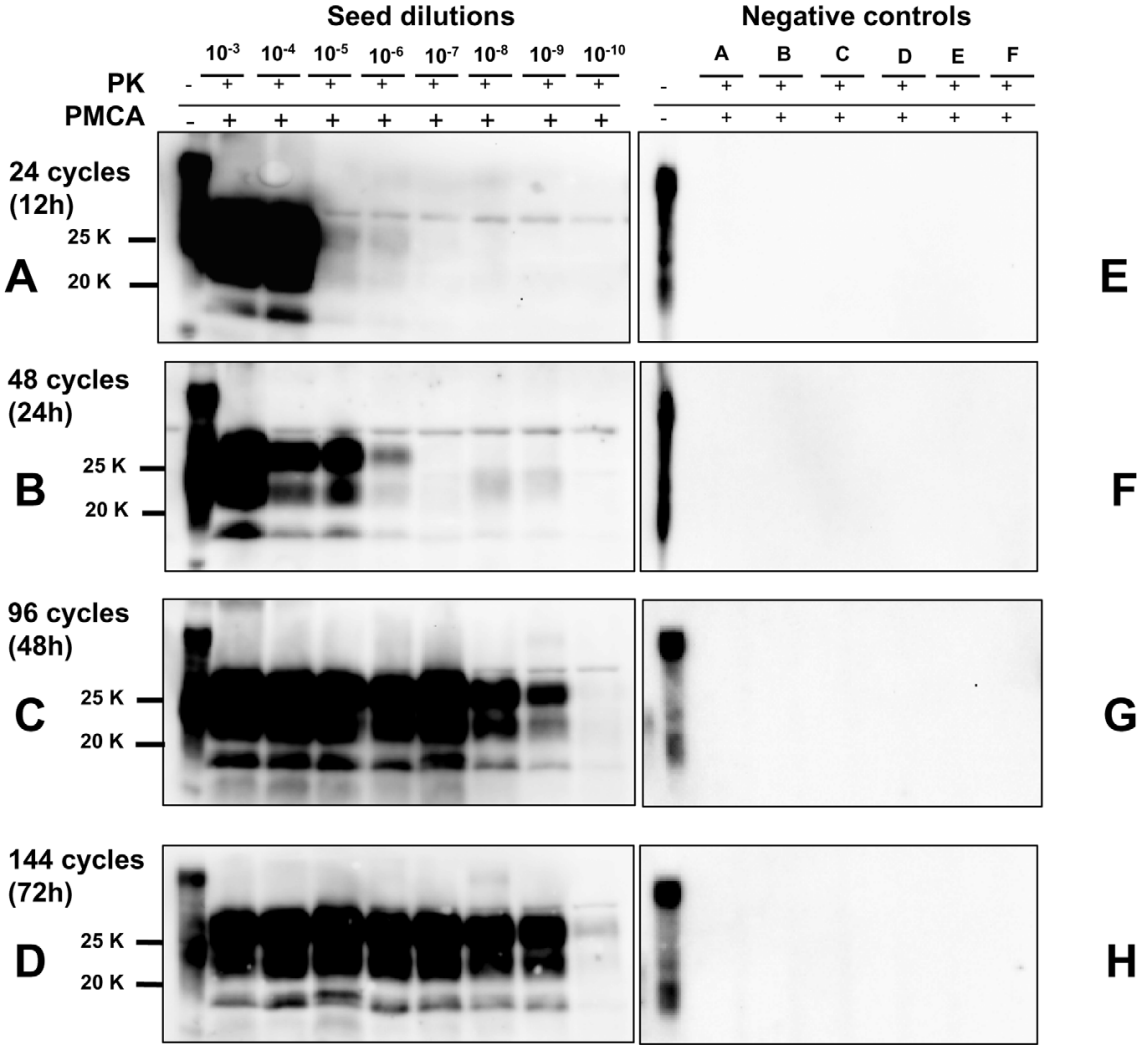
Kinetics of PrP^Sc^ amplification during a single round of PMCA. Logarithmic dilutions of v586 seed from 10^−3^ to 10^−10^, were prepared in quadruplicate using M109M vole substrate. Samples were amplified for a single round of PMCA together with unseeded negative controls. The level of amplification was evaluated by western blot in seeded and unseeded samples after 12 hours (panels A and E), 24 hours (panels B and F), 48 hours (panels C and G) and 72 hours (panels D and H). Western blot was probed with SAF84 primary antibody.

**Figure 5:**
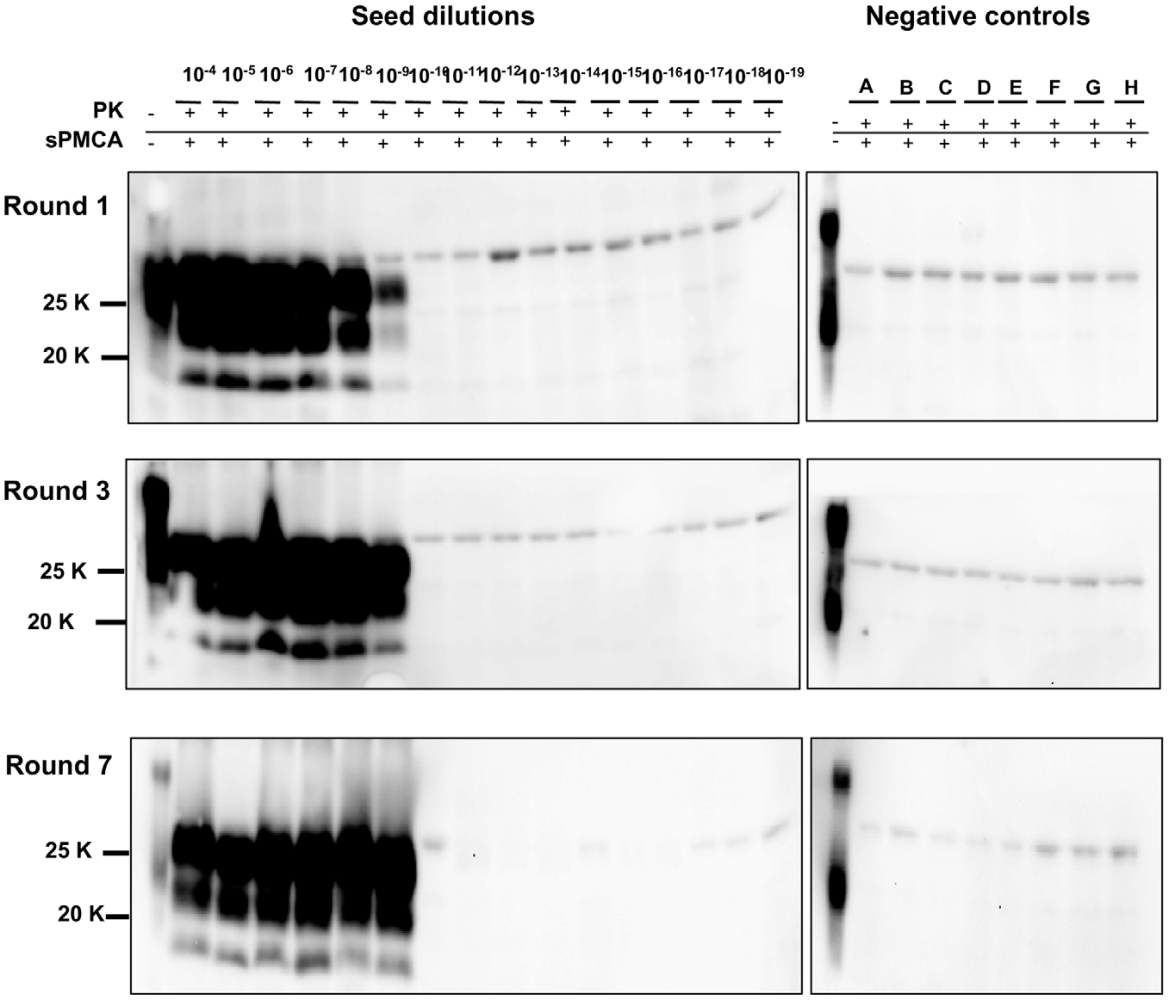
Limit of detection of v586 using saPMCA. Logarithmic dilutions of v586 seed from 10^−3^ to 10^−18^ were prepared using M109M vole substrate. and were amplified for 7 consecutive 48-hour rounds of saPMCA ( = 96 cycles of incubation/sonication), together with 8 unseeded negative controls (A to H). Positive amplification reached 10^−9^ dilution after the 1^st^ round. In the subsequent rounds all dilutions up to 10^−9^ continued to be positive while negative controls and dilutions from 10^−10^ to 10^−18^ remained negative. Western blot was stained with SAF84 primary antibody.

In order to achieve the best conditions for high amplification efficiency, which is useful in sensitive diagnostic tests, we investigated the conditions that could lead to the highest sensitivity in a limited number of rounds using 0,5 mL screw-cap vials. This was done by amplifying dilution curves of v586 inoculum and evaluating the level of amplification obtained after 12, 24, 48 and 72 hours of PMCA using standard settings (Figure 4). By increasing the duration of PMCA from 24 to 48 hours, we observed a 3-log improvement in PrP^Sc^ amplification, making it possible to increase considerably the overall sensitivity attainable in a single round. Further increasing the duration to 72 hours did not, however, seem to be useful, since the amplification level did not substantially increase. This was further investigated by testing the ability of 48 h-old substrates (previously treated with 48 hours of incubation/sonication cycles) to support PrP^Sc^ amplification. The failure of 48 h-old substrates to amplify PrP^Sc^ (Figure S9) confirmed that the ability of substrates to support amplification dramatically decreases after 48 hours of PMCA. Single-round 48-hour PMCA was thus set up and a limit of detection ranging from 10^−9^ to 10^−10^ was observed when the experiments were repeated several times using different v586 seeds preparations and different substrates. We then attempted the serial 48-hour PMCA in order to investigate the limit of detection of v586 under these new conditions. As shown in Figure 5, the level of amplification reached after the first round did not increase further in successive PMCA rounds. Importantly, negative controls and dilutions exceeding the limit of 10^−9^ remained negative up to the seventh PMCA round. Therefore, by introducing 0,5 mL screw-cap vials and 48-hour PMCA rounds, cross-contamination was efficiently controlled while ultra-high sensitivity was maintained for many consecutive rounds.

Since then we have performed numerous saPMCA experiments using the same working conditions. The appearance of spurious PrP^Sc^ signals was observed in only two out of more than 500 samples that were submitted to saPMCA. One of the two samples was an unseeded negative control, the other was a10^−15^ dilution of v586. The PrP^Sc^ of these samples preserved the same biochemical properties as the v586 seed used in the experiments. This clearly indicates that although cross-contamination in the sonicator can be effectively prevented by screw-cap vials, contamination during manipulation of samples can still lead to rare cross-contamination events, as often happens during ultrasensitive diagnostic tests.

## Discussion

In the present study we describe the results of a large series of PMCA experiments under strictly controlled conditions which highlight the drawbacks and potential of PMCA when ultrasensitive detection is desired and propose technical improvements to increase the robustness of the technique.

This work was stimulated by observing how the extraordinary potential of PMCA is accompanied by a disturbing production of infectious PrP^Sc^ in unseeded reaction tubes, which initially led us to hypothesise the occurrence of “spontaneous” or *de novo* generation of prions.

The *de novo* generation of prions agrees with the prion theory, which postulates that sporadic TSEs may originate from the stochastic occurrence of spontaneous PrP^C^ conversion into PrP^Sc^. Several lines of evidence support this hypothesis. Infectious prions have been generated from β-rich recombinant mouse PrP(89–230) [5]. Aguzzi and colleagues reported the spontaneous generation of prions in transgenic mice carrying a mutated prion gene (S170N, N174T) [32]. They also observed prion infectivity in mice over-expressing wild type PrP (*tg*a*20* line) after inoculation of brain homogenates from extremely aged, uninoculated mice of the same line. The spontaneous occurrence of prions in cell cultures has recently been reported by Edgeworth and colleagues [33].

As PMCA is able to reproduce *in vitro* several aspects of prion biology, it appears theoretically to be an appropriate technique for modelling the *de novo* generation of prions, making it this field of investigation very attractive.

However, the hypothesis that prions can be generated *de novo* is not easy to refute experimentally and - given that *de novo* prions are postulated to have the same characteristics and properties as other prions – there is no way to distinguish prions generated *de novo* from potential contaminants. Nonetheless, it was recently reported that full-length recombinant prion protein, that had been converted into the cross-beta-sheet amyloid form and subjected to annealing, gave rise to a disease with clearly distinctive phenotype from archetypal 263K, upon serial transmission in hamster [7]. However, drawing an *a posteriori* distinction between prions putatively generated *de novo* and prions from other sources recognised as putative contaminants is hampered by the potential of prions to mutate, both *in vivo* [34] and *in vitro* [35]. In our experiments, supposedly *de novo* prions generated in vole PMCA were not easily distinguishable from other conventional prion strains (Figures S2, S3). The only way to obtain direct evidence of the *de novo* generation of prions is by establishing experimental conditions in which the occurrence of contamination can be excluded categorically. Since prions are not thought to be ubiquitous contaminants, the use of rigorously prion-free procedures appears adequate to achieve these conditions.

We performed several unseeded PMCA experiments with hundreds of samples processed and analysed over more than 6 months in absolutely prion-free conditions. Different PMCA settings and several substrates from many voles of different ages were used. No evidence of *de novo* generation of prions was obtained, despite the high levels of sensitivity reached.

Our results appear to be in contrast with those obtained by other groups. The *de novo* generation of prions during PMCA experiments has been reported by several authors. Saa et al. [25] reported the spontaneous generation of PrP^Sc^ in unseeded PMCA reactions, especially when more than 10 rounds of standard PMCA were performed. However, they were unable to state conclusively whether the newly generated PrP^Sc^ was the result of cross-contamination or *de novo* generation. Deleault et al. [4] and Thorne and Terry [26] obtained putative *de novo* prions in unseeded PMCA reactions only when a synthetic polyanion (PolyA) was added, but not in its absence. Barria et al. [27] reported that *de novo* generation was not seen under standard PMCA conditions but could be obtained only by prolonging the duration of PMCA rounds from 3 to 5 days. Recently Wang et al. [6] observed the appearance of infectious prions from unseeded samples after numerous consecutive rounds of saPMCA in a novel PMCA system based on purified recombinant PrP, a synthetic anionic lipid and liver RNA, but not when normal mouse brain was used.

The PMCA conditions that allow prions to be generated *de novo* are thus still unclear. Interestingly, on the basis of the results reported by Barria and co-authors [27], who observed *de novo* prions only using very extended PMCA rounds, it could be concluded that the spontaneous generation of prions does not follow the same replication model as the seeded amplification. *De novo* prions would seem to be generated under conditions in which PrP^C^ and other potential co-factors contained in the substrate have lost their ability to support the seeded amplification. Both our (Figure S9) and Soto's results [18] indeed demonstrate that the substrates lack their potential to support amplification after 72 hours of sonication/incubation.

The key factor in demonstrating that prions have been generated *de novo* is the stringency of measures taken to avoid contamination. Unfortunately, details on measures taken to control cross-contamination between seeded and unseeded samples were not always presented. Although the problem of false positives has been addressed in recent papers [36], [25], Deleault et al. and Barria et al. [4], [27] reported that prions were generated *de novo* in conditions of extremely reduced possibility of cross contamination. Nonetheless, Barria et al [27] and Wang et al [6] concluded that cross-contamination could not be entirely ruled out.

The efficiency of PMCA is variable, depending on the combination of seed and substrate. Sonicator settings are often adapted to reach maximum efficiency [18]. Considering that one of the main objectives of this study was to investigate the spread of contamination during saPMCA, we decided to adopt v586 as infected inoculum for seeded experiments because it was a contaminant in origin (see Methods). After seeding vole substrate with v586 strain amplification proved extremely efficient. The limit of detection, observed in several independent experiments, ranged between 10^−9^ and 10^−10^ of the infected brain homogenate in a single 48-hour round of PMCA. Additional rounds did not improve the overall sensitivity. These data are in agreement with the efficiency reported for mouse-adapted scrapie and BSE, which is the highest reported in the literature [37]. Results of the limiting dilution experiments showed that 10^−10^ is the highest dilution of the inoculum able to trigger a positive amplification, corresponding approximately to 6 femtoliters of infected brain homogenate. Similar levels of contamination are not easy to control, especially for a procedure that is performed using equipment originally developed for other purposes. We have also observed cross-contamination when working with PMCA substrates from other species, such as mice (data not shown), although it was observed at a lower level and after an higher number of PMCA rounds. This might be due to the lower amplification efficiency we have observed with mouse-derived compared to vole-derived substrates. When ultrasensitive amplification levels are reached, such as those attained with vole brains as substrates, the risk that minimal inadvertent contamination of reactions may artificially initiate amplification is extremely high. These findings suggest the need to critically reassess previous reports of supposedly de novo prion generation obtained by standard PMCA setting.

In experiments using only unseeded samples, we determined that the equipment used (pipettes, sonicator, thermal bath) and the working environment (laminar flow hood, bench) are not direct sources of contamination, provided that general safety measures are adopted. It is unlikely that they can release prions potentially acquired during previous experiments at levels able to trigger amplification in subsequent PMCA reactions. In contrast, direct or indirect cross-contamination easily occurs between seeded and unseeded samples when these are manipulated and processed at the same time. The presence of high numbers of seeded tubes in the sonicator and the progressive and dramatic increase in amplified products during serial rounds further increase the risk.

Sonication pulses violently shake the samples. The generation of aerosol and micro-particles within the reaction tubes during sonication probably represents a key critical factor by enormously increasing the risk of contamination of the rim of the tube and of material leakage at the opening of the tubes. The use of Multiply-Safecup Biosphere tubes radically improved the handling of samples and, thanks to both the screw cap and the volume limitation system radically reduced the risk of leakage. The preservation of the same amplification efficiency using larger tubes with thicker walls and other significant differences compared with PCR tubes, without the need to modify the sonication parameters, demonstrates that PMCA is capable of coping well with significant changes in the operating environment.

In the course of the study we observed a dramatic decrease in false positive results from the moment we adopted the screw-cap tubes. This provides strong evidence that false positive results actually derive from cross-contamination events which can be efficiently prevented by tighter closure of the sample tubes. Spurious results were observed even with the highest level of safety measures, but these were very rare and comparable to those observed with other techniques. We cannot exclude the possibility that some of these events actually concealed the *de novo* generation of prions, but while we cannot dismiss alternative explanations, the occurrence of inadvertent contamination still appears the most appropriate and scientifically sound interpretation. This is further strengthened by the unique molecular signature observed throughout our study, similar to the original v586 seed. The few spurious results that we observed after the adoption of screw-cap tubes occurred after several rounds of serial PMCA, and could be tentatively explained by inadvertent contamination during manipulation of the tubes for seeding successive rounds of serial PMCA. In this respect, methods able to increase the sensitivity of PMCA, as recently reported by Gonzalez-Montalban and colleagues [38], would reduce the number of serial passages needed to achieve ultrasensitive detection of PrP^Sc^ and, conceivably, would also be valuable for preventing cross-contamination events.

The results of the present study certainly do not exclude the possibility that, in different experimental conditions, prions can be generated *de novo*. They provide a serious warning that contamination can very easily occur when ultrasensitive levels of detection are reached and that extreme caution is needed to avoid the over- or misinterpretation of results when delicate issues such as the *de novo* generation of prions are explored.

## Methods

### Ethics statement

The research protocol was approved by the Service for Biotechnology and Animal Welfare of the Istituto Superiore di Sanità and authorized by the Italian Ministry of Health, according to Legislative Decree 116/92, which implemented the European Directive 86/609/EEC on laboratory animal protection in Italy. Animal welfare was routinely checked by veterinarians from the Service for Biotechnology and Animal Welfare.

### Preparation of substrates

Substrates were prepared from male and female bank voles of different ages, carrying either M109M or I109I PrP genotypes. Unless indicated otherwise (see Tables 1 and S1), the animals were 2–4 months old. Voles were sacrificed using carbon dioxide and immediately perfused with phosphate-buffered saline (PBS) plus 5 mM ethylendiaminetetraacetic acid (EDTA). Immediately after perfusion, the brains were dissected and frozen at −80°C. 10% brain homogenates (weight/volume) were prepared in conversion buffer (PBS 1x, pH 7,4; 0,15 M NaCl; 1% Triton X-100) with the Roche Complete Protease inhibitor cocktail (1 tablet in 50 mL conversion buffer), using new and dedicated glass potters. Substrates were divided into aliquots and either used or stored at –80°C immediately after homogenisation. *De novo* generation experiments (Tables 1 and S1) were carried out using substrates from individual voles while for seeded experiments (see Results), requiring larger amounts of substrate, we prepared homogeneous pools of substrates by mixing together 5 individual substrates from voles of 2–4 months of age. Preparation and storage of substrates were carried out in a laboratory never previously used for prion research, using equipment specifically dedicated and maintaining rigorously prion-free conditions.

### Preparation of the inoculum for PMCA

The vole-passaged spontaneous M109M strain B (see results), indicated in the main text as v586, was used as prion inoculum for seeded PMCA experiments. Brain tissue from terminally affected M109M voles after second passage of M109M strain B (see Table S2) was homogenised in PBS (10% w/v) containing Complete Protease inhibitor cocktail (Roche) using disposable Teflon pestles directly in 1.5 mL Eppendorf tubes. Following preparation the brain homogenate was divided into aliquots and stored at −20°C.

#### Preparation of dilution curves of the inoculum

v586 brain homogenate was serially diluted in vole substrate. Curves were prepared as follow: 20 µl of inoculum were added in a 1,5 mL Eppendorf tube containing 180 µl of substrate, followed by 10-fold serial dilutions in 1,5 mL Eppendorf tubes containing 180 µl of substrate. At each dilution, inoculum and substrate were mixed by gently pipetting 50 times paying careful attention to change the tip of the pipette at each dilution step. Finally, 60 µl of each dilution were introduced into PMCA tubes for the first round of amplification. Samples were handled under a laminar flow hood, using dedicated pipettes and double filter tips, carefully avoiding leakages of material during the manipulation of tubes. The use of a vortex instead of pipetting is not recommended as, in our experience it has occasionally been associated with the appearance of spurious positive PrP^Sc^ signals after amplification.

### Serial automated PMCA (saPMCA)

PMCA was performed according to the protocol of Castilla et al.[18]. Amplification was performed in a total volume of 60 µl. Tubes containing the reaction mix were incubated at 37°C, placed in a disk-shaped rack on the microplate horn of the sonicator (Misonix S3000). The horn was placed in a gravity oven. Water in the horn was circulated in a thermal bath at a constant temperature of 37°C . The standard sonication programme consisted of 30 second sonication pulses every 30 minutes for 24- or 48-hour periods. In particular cases single parameters of PMCA were modified (see Table 1) in order to investigate different conditions of amplification. At the end of each round, the PMCA material was gently span and diluted 1∶10 in fresh substrate ready for a new amplification step. Passages of seeded experiments were carried out in a laminar flow hood, using double filter pipette tips (Eppendorf Dualfilter T.I.P.S.). During this study two kinds of tube were used to contain PMCA reactions: we began working with 0.2 mL tubes for PCR (NUNC, cat. 248161), then continued with 0,5 mL screw cap Multiply- Safecup (Sarstedt, cat. 72.733.200) (see Results).

### Additional procedures to minimise contamination

Access to the laboratory was restricted to authorised personnel and standard operating procedures were adopted for the use of laboratory equipment and sample manipulation. All personnel wore disposable coats, shoe covers and gloves. The working surfaces and equipment were cleaned with NaOH or NaClO after each experiment. These measures were further strengthened by changing the water of the sonicator circuit and decontaminating the plastic holder and horn cap by immersion in bleach after each round.

### Electrophoresis and immunoblotting

20 µL of PMCA material were digested with 100 µg/mL proteinase K (pK) (Sigma-Aldrich). Samples were shaken at 900 rounds per minute (rpm) for one hour at 37°C. Digestion was then blocked by adding 2 µL of 10 mM phenylmethylsulfonyl fluoride (PMSF) (Pierce Biotechnology - Thermo Fisher Scientific); samples were then incubated for 5 minutes at 4°C. Ten micro-litres of NuPAGELDS Sample Buffer (4X) and 3 µL NuPAGE Sample Reducing Agent (10X) were added to each tube and samples were denatured by incubation at 90°C for 10 minutes. *Western blot analysis*: 12 µL of pK-digested PMCA samples were resolved by SDS-PAGE on a 10% Tris-glycine gel and transferred to PVDF. The membrane was blocked with 1% powdered skim milk in PBS for one hour at room temperature and incubated with mouse anti-PrP monoclonal antibody SAF84 (Cayman Chemical). After washing, the membrane was incubated with secondary HRP-conjugated antibody (ImmunoPure Peroxidase Conjugated Goat Anti-Mouse IgG (H+L) Pierce Biotechnology - Thermo Fisher Scientific) and signals were detected using SuperSignal West Femto Maximum Sensitivity Substrate (Pierce Biotechnology - Thermo Fisher Scientific). Chemiluminescence was detected with the VersaDoc imaging system (Bio-Rad). All measurements were performed with QuantityOne software (Bio-Rad).

## Supporting Information

Figure S1 **Putative** ***de novo*** **vole PrP^Sc^ types obtained by vole sa-PMCA.** Samples from 23 different unseeded experiments of vole saPMCA (see Table S1) were studied by western blot. Based on the differential electrophoretic mobilities, two distinct PrP^Sc^ types (named A and B for the high and low MW types, respectively) were recovered from both vole genotypes. Putatively *de novo* strains A and B were further passaged for up to 10 serial round of PMCA maintaining their distinctive electrophoretic mobility. Western blot was stained with D18 primary antibody.
(TIF)

Figure S2 **Biochemical comparison between the putative** ***de novo*** **PrP^Sc^ types and vole-adapted prions.** Prototypical vole TSEs (SSBP1 and SS8, derived from sheep scrapie, sCJDMV1 and sCJDMV2 derived from human, cattle-derived BSE and mouse-derived 301C) obtained after serial *in vivo* passages in voles, were compared with the four putative *de novo* PrP^Sc^ types obtained *in vitro.* PrP^Sc^ from all samples was digested with PK and analysed by WB using two different antibodies, SAF84 and 12B2. These two antibodies are used for discriminating PrP^res^ types according to the N-terminal cleavage by PK. Indeed, SAF84 recognizes a C-terminal epitope and binds all kinds of vole prion strains, while 12B2 binds an epitope in the region differentially cleaved by PK. The figure shows that putative *de novo* PrP^Sc^ types M109MA and I109IA are similar to scrapie-like vole prions. The same types are also similar to the sCJD MV1-derived vole prion. On the other hand the *de novo* PrP^Sc^ types M109MB and I109IB have a molecular weight similar to BSE and sCJD-MM2-derived vole prions, and are not recognized by 12B2.
(TIF)

Figure S3 **Biochemical comparison between putative** ***de novo*** **PrP^Sc^ types and PMCA-amplified vole prions.** Vole-adapted prion strains derived from human (sCJDMM1), sheep (SS8 and SSBP1), cattle (BSE) and mice (301C and 301V) were amplified *in vitro* by 15 rounds of saPMCA using vole substrate and were compared with the four putative *de novo* PrP^Sc^ types obtained in unseeded PMCA reactions. Replica blots were stained with mAbs SAF84 and 12B2 (see figure S2). The *de novo* PrP^Sc^ types A from M109M and I109I show biochemical features similar to PMCA-passaged scrapie strains and sCJD-MM1, while *de novo* PrP^Sc^ types B are similar to PMCA-passaged BSE and BSE-derived strains (301C and 301V).
(TIF)

Figure S4 **Biochemical comparison between putative** ***de novo*** **prion strains after** ***in vivo*** **passage in voles.** Representative discriminatory WB (see figure S2) from PK digested brain homogenates of vole-passaged putative *de novo* prion strains (M109MA and M109MB). PrP^Sc^ from scrapie-like vole-adapted ME7 was used as control. The figure shows that scrapie-like and BSE-like biochemical properties were preserved after *in vivo* passage of strains A and B, respectively.
(TIF)

Figure S5 **PrP^Sc^ deposition patterns in vole-passaged putative** ***de novo*** **prion strains. A)** PET-blot analysis with SAF84. In M109MA PrP^Sc^ deposition was predominant in the cortex, caudate-putamen and gyrus dentate compared to M109MB infected voles. Moreover, the hypothalamus was much more involved in M109MA than in M109MB. **B**) Immunohistochemistry performed with polyclonal rabbit Ab R486 shows distinct PrP^Sc^ deposition patterns in M109MA and M109MB. Punctuate PrP^Sc^ deposition associated with conspicuous vacuolization was found in the medial geniculate nucleus of M109MA, whereas in M109MB an astrocytic pattern was observed. In the parietal cortex of M109MA perivascular and punctuate patterns were evident, while M109MB showed an astrocytic pattern.
(TIF)

Figure S6 **Lesion profiles in voles infected with seeded and unseeded PMCA products. A**) Lesion profiles in voles after transmission of M109MA and M109MB show distinct patterns of spongiform degeneration. **B**) Comparison of the lesion profiles in M109MA and SS8 show partial overlapping. **C**) Comparison of the lesion profiles in M109MB, 301C and 301V show near complete overlapping, particularly between M109MB and 301C. Brain-scoring positions are medulla (1), cerebellum (2), superior colliculus (3), hypothalamus (4), thalamus (5), hippocampus (6), septum (7), retrosplenial and adjacent motor cortex (8), and cingulate and adjacent motor cortex (9).
(TIF)

Figure S7 **A test of the efficiency of PMCA.** Two-fold serial dilution was prepared from 1∶100 to 1∶12800 using v586 inculum and vole M109M substrate. Samples were amplified for a single round of 24 hours (48 cycles of sonication/incubation). The first lane shows the unamplified “frozen” 1∶200 dilution and the second lane shows the dilution curve after PMCA. Western blots were probed with SAF84 primary antibody.
(TIF)

Figure S8 **Screw-cap tube for PMCA.** Containment of PrP^Sc^ during saPMCA was improved by using Sarstedt 0.5 mL Multiply-Safecup with 100 µl volume limitation. This tube is easy to handle, the volume limiter confines reaction mix to the bottom of the vial, where it is better exposed to the effect of the ultrasound, keeping the substrate away from the rim of the tube. Reaction sensitivity is comparable with 0,2 mL PCR tubes, while control of cross-contamination is much more efficient.
(TIF)

Figure S9 **Evaluating the ability of substrate to amplify PrP^Sc^ after 48 h of PMCA.** Logarithmic dilutions of v586 seed were prepared from 10^−4^ to 10^−10^ using either: freshly prepared M109M vole substrate (**A**) or a substrate carrying the same PrP genotype previously submitted to repeated cycles of incubation/sonication for 48 hours (**B**). Dilutions were submitted to a single round of 48 hours of PMCA. Western blots were probed with SAF84 primary antibody.
(TIF)

Table S1 **Supposedly *de novo* PrP^Sc^ appearance in unseeded PMCA reactions using bank vole substrates.**
(DOC)

Table S2 **Transmission of putative *de novo* vole prion strains by intra-cerebral inoculation in voles.**
(DOC)

Table S3 **Comparison of seeded and unseeded saPMCA products by intra-cerebral inoculation in voles.**
(DOC)

Text S1 **Supporting results.** Early experimental evidences indicated that the appearance of PrP^Sc^ in unseeded substrate samples was frequent after a variable number of saPMCA rounds. This finding was repeatedly observed in several different independent experiments and using numerous vole substrates (Table S1). PrP^Sc^ isolates from unseeded experiments were studied by western blot. Based on the differential electrophoretic mobilities, two distinct PrP^Sc^ types (named A and B) were recovered from M109M and I109I vole genotypes. Putatively *de novo* strains A and B were further passaged for up to 10 serial round of PMCA maintaining their distinctive electrophoretic mobility. (Figure S1). The PrP^Sc^ types, that were supposed to be of spontaneous origin, have been intensively studied using methods available for TSEs strain typing (Figures S2, S3, S4, S5, S6 and Tables S2 and S3).
(DOC)

Text S2 **Method section for supporting Figures and Tables.**
(DOC)
