# Supplementary figures and images for: Ultra-Efficient PrPSc Amplification Highlights Potentialities and Pitfalls of PMCA Technology
Source: PLoS Pathog. 2011 Nov 17;7(11):e1002370. doi: 10.1371/journal.ppat.1002370 (PMC3219717; doi:10.1371/journal.ppat.1002370)

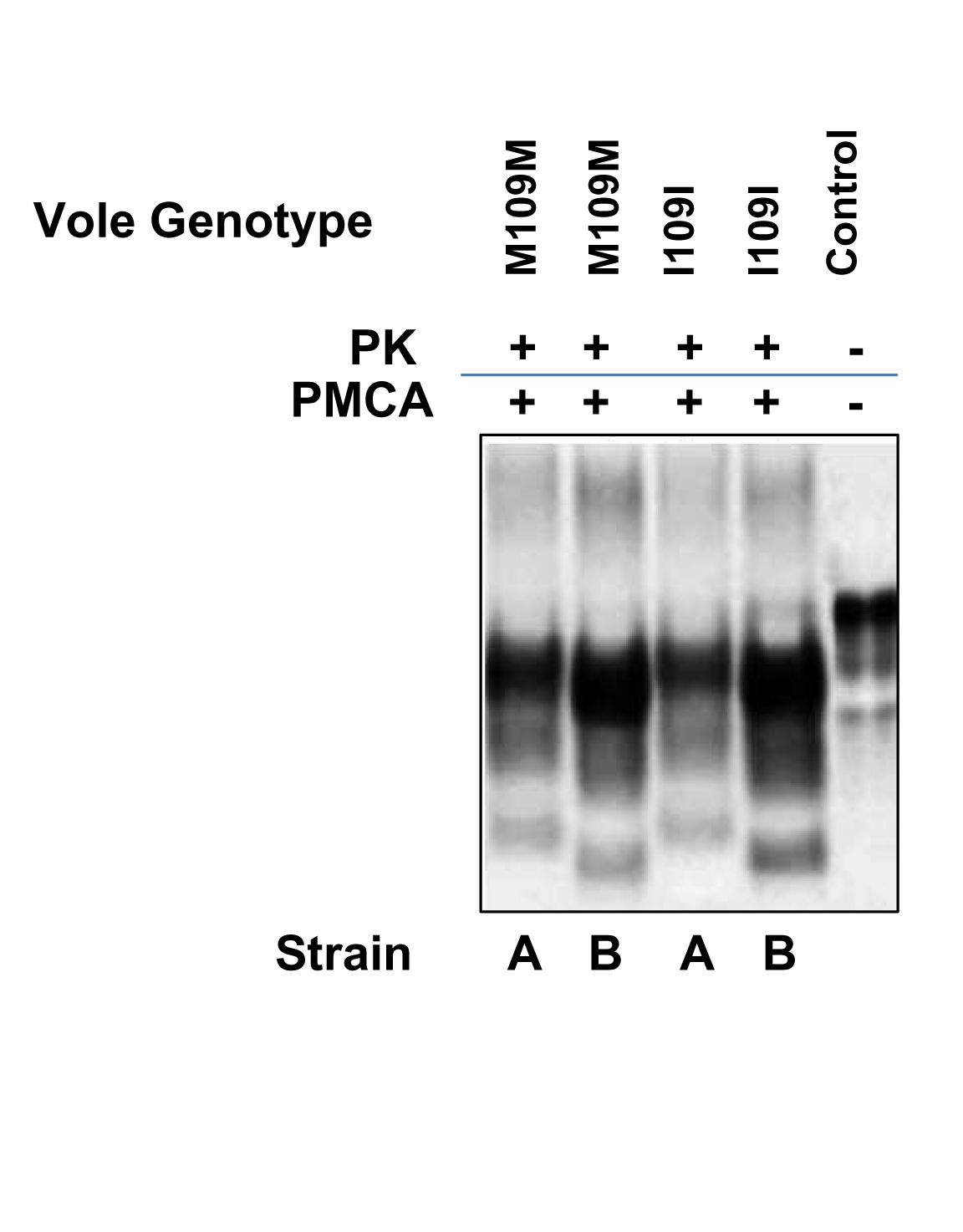

Supplement: Figure S1 — Putative de novo vole PrPSc types obtained by vole sa-PMCA. Samples from 23 different unseeded experiments of vole saPMCA (see Table S1) were studied by western blot. Based on the differential electrophoretic mobilities, two distinct PrPSc types (named A and B for the high and low MW types, respectively) were recovered from both vole genotypes. Putatively de novo strains A and B were further passaged for up to 10 serial round of PMCA maintaining their distinctive electrophoretic mobility. Western blot was stained with D18 primary antibody. (TIF) [file ppat.1002370.s001.tif]

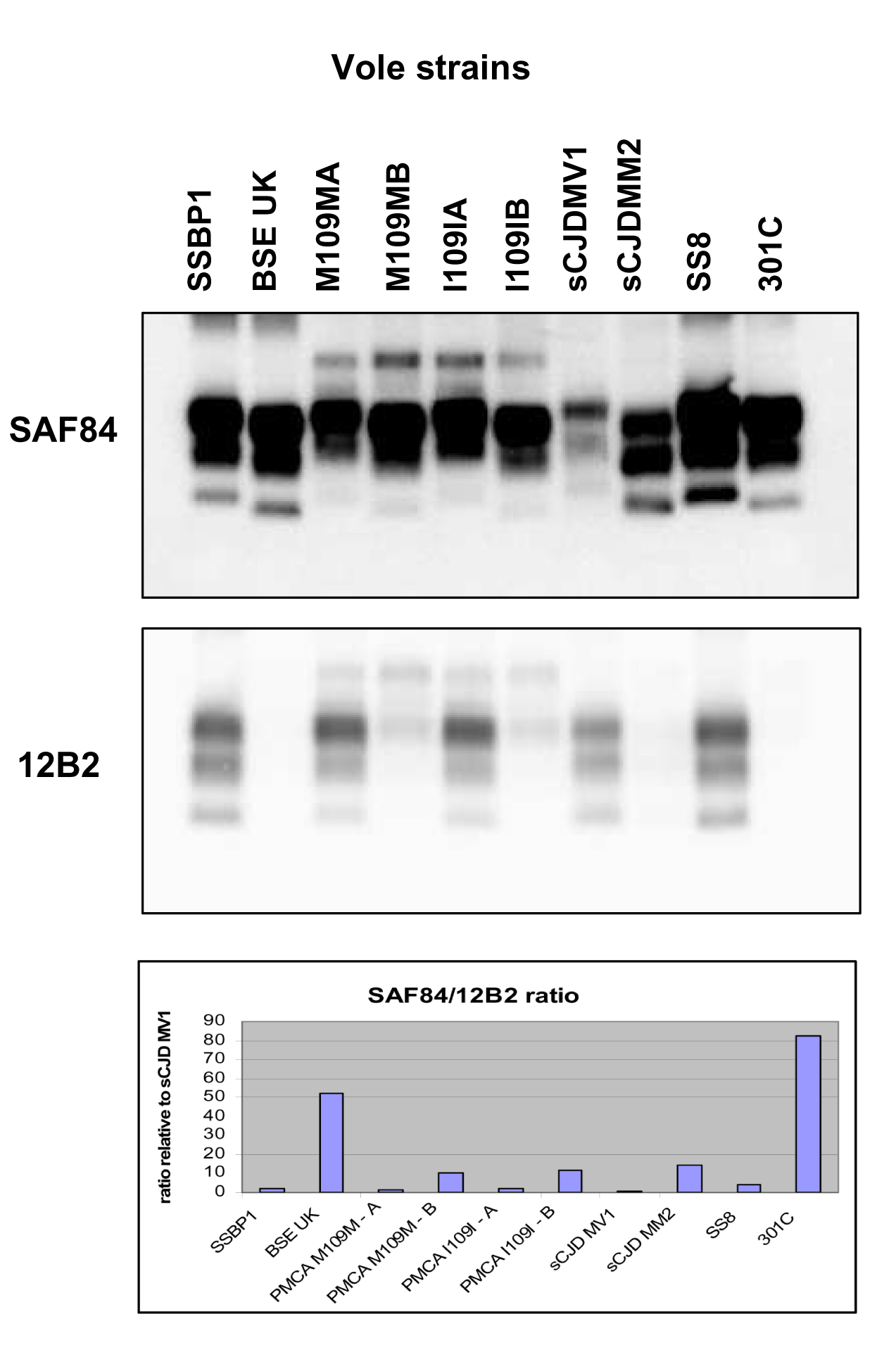

Supplement: Figure S2 — Biochemical comparison between the putative de novo PrPSc types and vole-adapted prions. Prototypical vole TSEs (SSBP1 and SS8, derived from sheep scrapie, sCJDMV1 and sCJDMV2 derived from human, cattle-derived BSE and mouse-derived 301C) obtained after serial in vivo passages in voles, were compared with the four putative de novo PrPSc types obtained in vitro. PrPSc from all samples was digested with PK and analysed by WB using two different antibodies, SAF84 and 12B2. These two antibodies are used for discriminating PrPres types according to the N-terminal cleavage by PK. Indeed, SAF84 recognizes a C-terminal epitope and binds all kinds of vole prion strains, while 12B2 binds an epitope in the region differentially cleaved by PK. The figure shows that putative de novo PrPSc types M109MA and I109IA are similar to scrapie-like vole prions. The same types are also similar to the sCJD MV1-derived vole prion. On the other hand the de novo PrPSc types M109MB and I109IB have a molecular weight similar to BSE and sCJD-MM2-derived vole prions, and are not recognized by 12B2. (TIF) [file ppat.1002370.s002.tif]

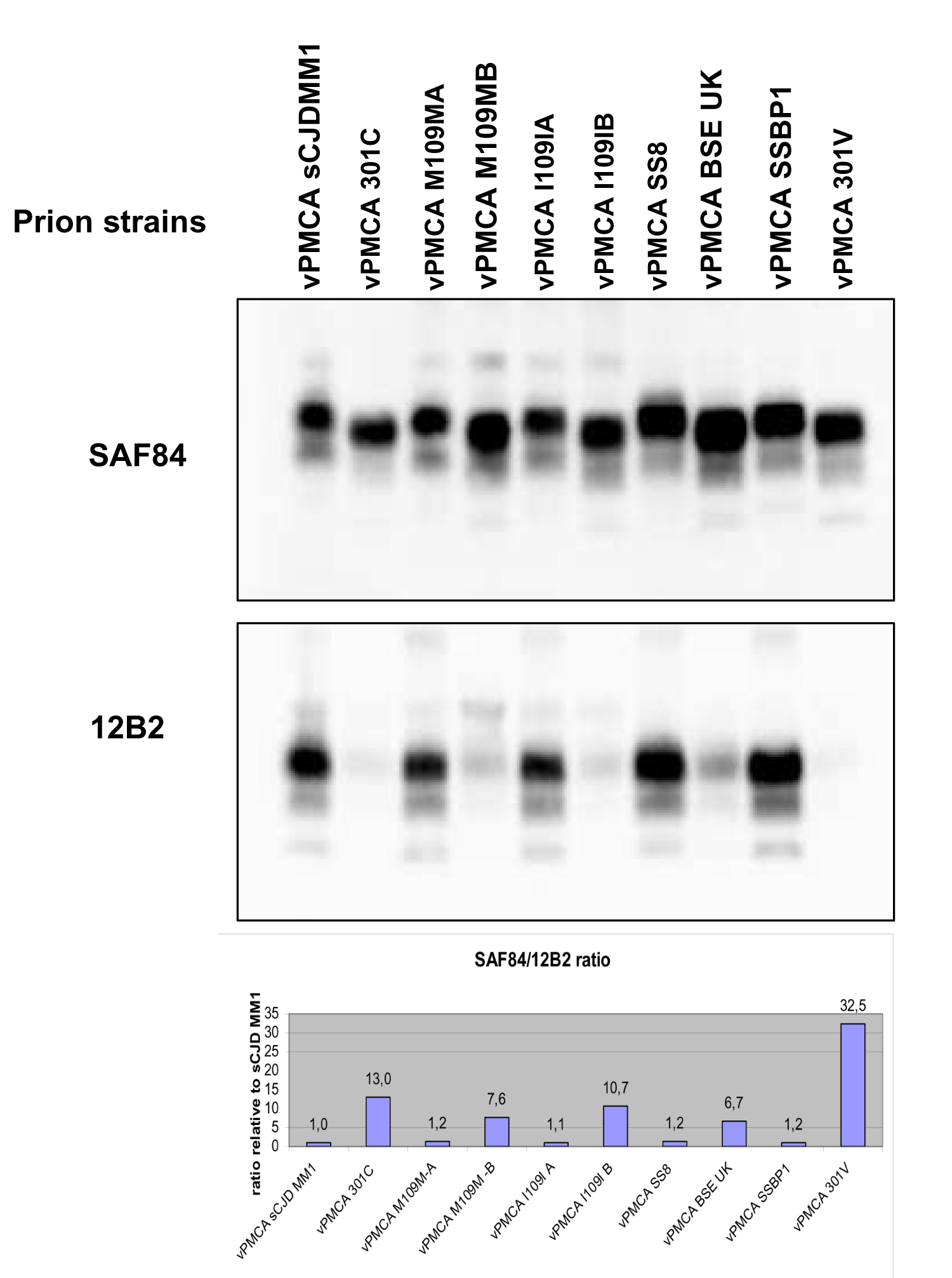

Supplement: Figure S3 — Biochemical comparison between putative de novo PrPSc types and PMCA-amplified vole prions. Vole-adapted prion strains derived from human (sCJDMM1), sheep (SS8 and SSBP1), cattle (BSE) and mice (301C and 301V) were amplified in vitro by 15 rounds of saPMCA using vole substrate and were compared with the four putative de novo PrPSc types obtained in unseeded PMCA reactions. Replica blots were stained with mAbs SAF84 and 12B2 (see figure S2). The de novo PrPSc types A from M109M and I109I show biochemical features similar to PMCA-passaged scrapie strains and sCJD-MM1, while de novo PrPSc types B are similar to PMCA-passaged BSE and BSE-derived strains (301C and 301V). (TIF) [file ppat.1002370.s003.tif]

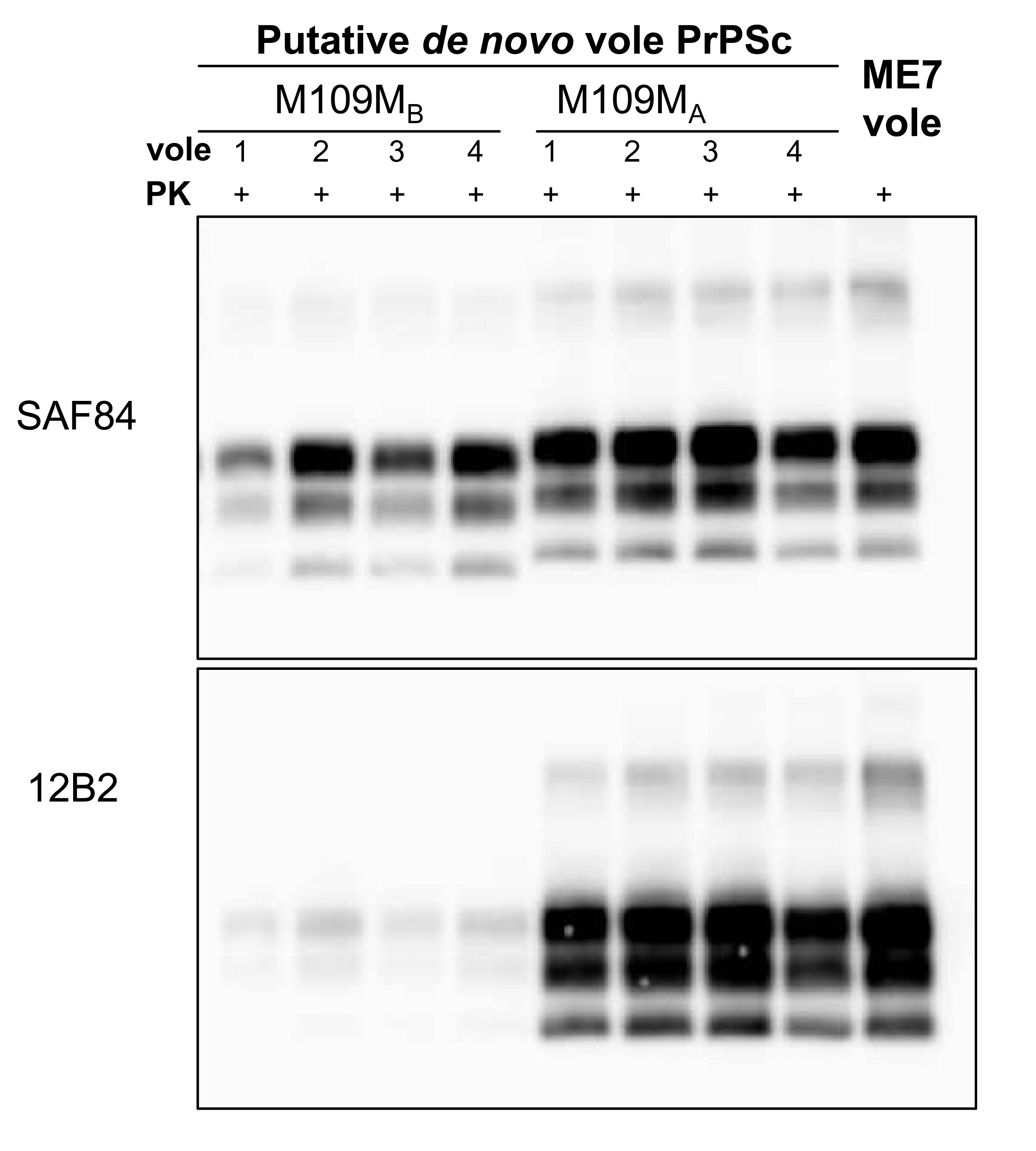

Supplement: Figure S4 — Biochemical comparison between putative de novo prion strains after in vivo passage in voles. Representative discriminatory WB (see figure S2) from PK digested brain homogenates of vole-passaged putative de novo prion strains (M109MA and M109MB). PrPSc from scrapie-like vole-adapted ME7 was used as control. The figure shows that scrapie-like and BSE-like biochemical properties were preserved after in vivo passage of strains A and B, respectively. (TIF) [file ppat.1002370.s004.tif]

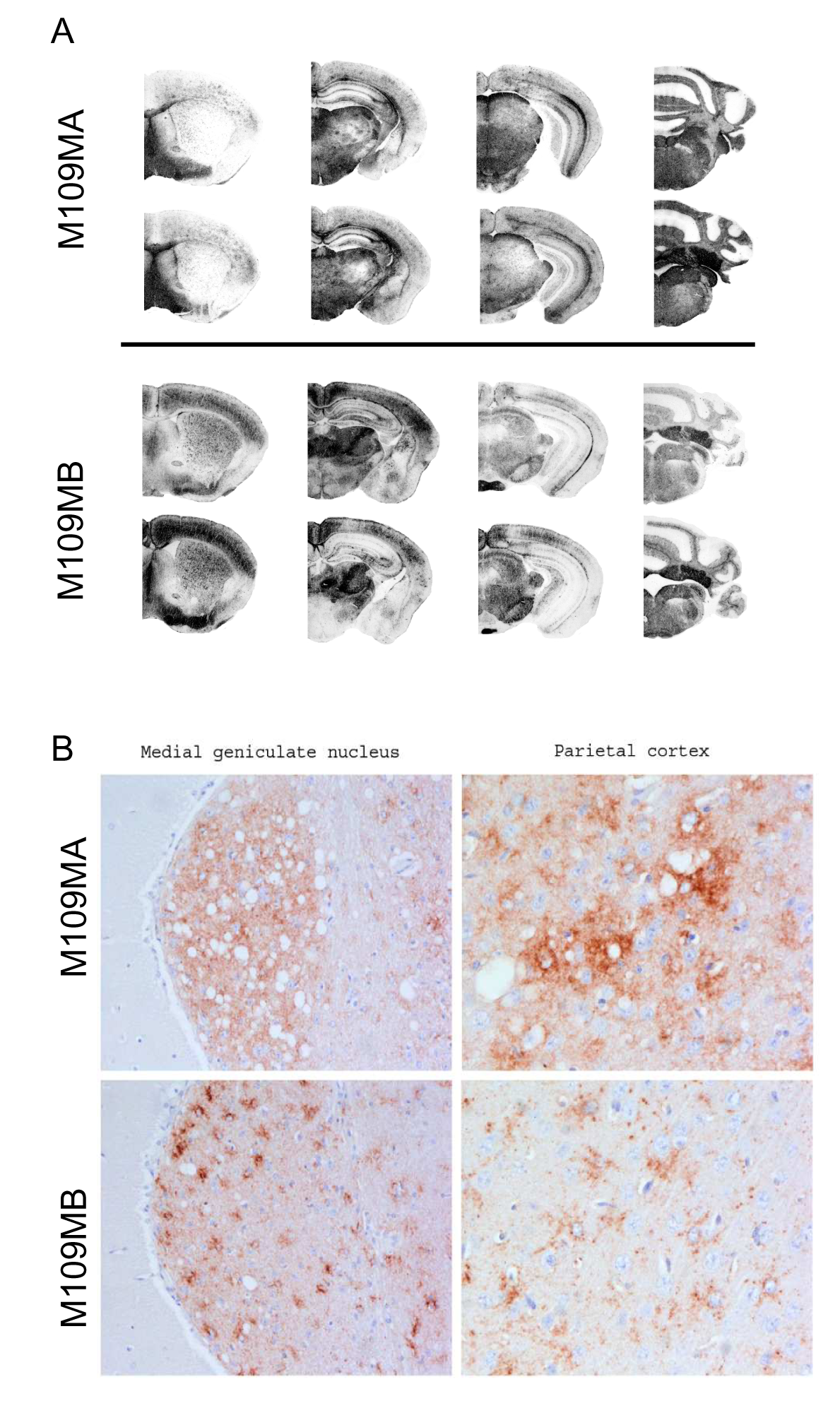

Supplement: Figure S5 — PrPSc deposition patterns in vole-passaged putative de novo prion strains. A) PET-blot analysis with SAF84. In M109MA PrPSc deposition was predominant in the cortex, caudate-putamen and gyrus dentate compared to M109MB infected voles. Moreover, the hypothalamus was much more involved in M109MA than in M109MB. B) Immunohistochemistry performed with polyclonal rabbit Ab R486 shows distinct PrPSc deposition patterns in M109MA and M109MB. Punctuate PrPSc deposition associated with conspicuous vacuolization was found in the medial geniculate nucleus of M109MA, whereas in M109MB an astrocytic pattern was observed. In the parietal cortex of M109MA perivascular and punctuate patterns were evident, while M109MB showed an astrocytic pattern. (TIF) [file ppat.1002370.s005.tif]

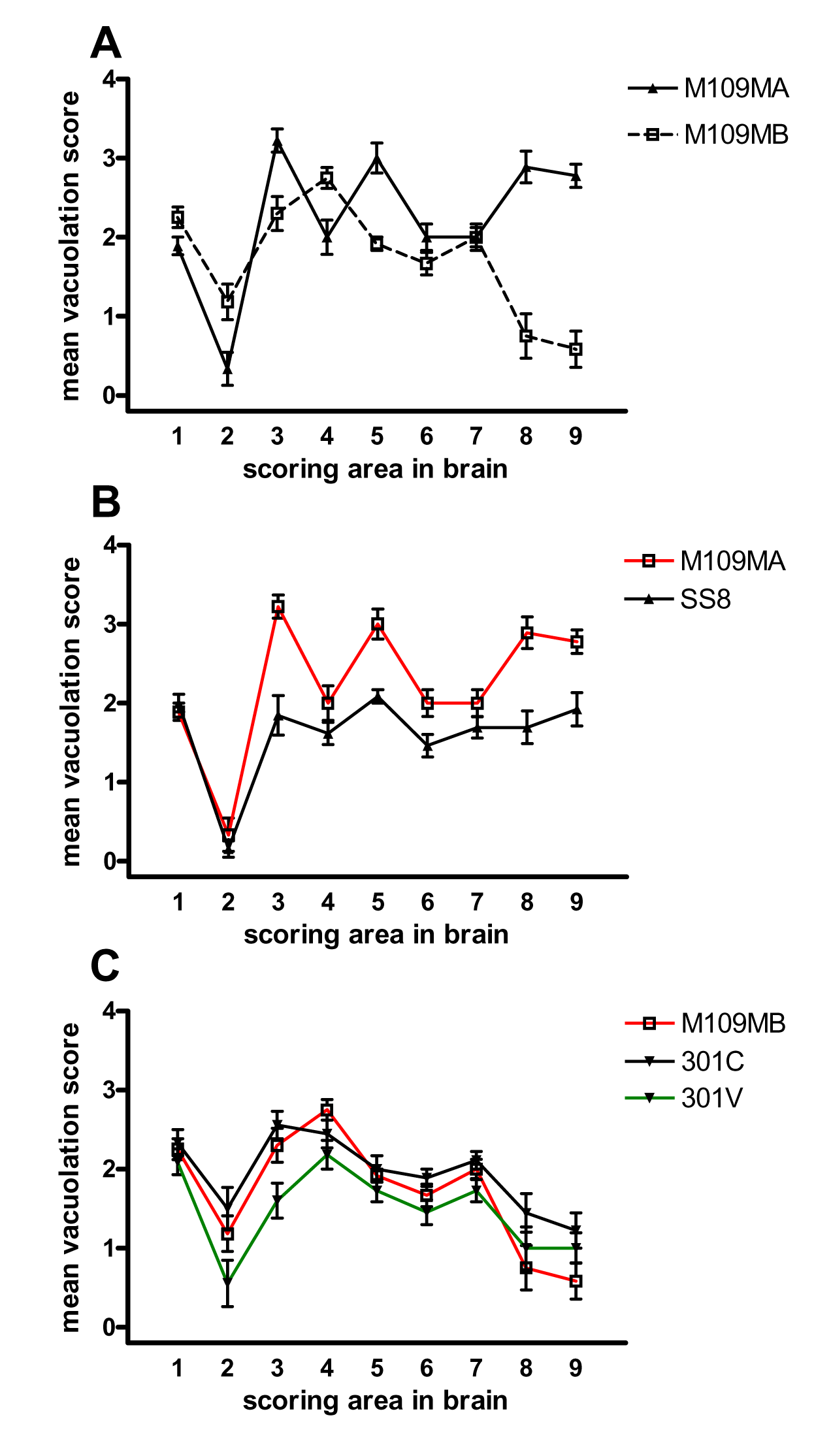

Supplement: Figure S6 — Lesion profiles in voles infected with seeded and unseeded PMCA products. A) Lesion profiles in voles after transmission of M109MA and M109MB show distinct patterns of spongiform degeneration. B) Comparison of the lesion profiles in M109MA and SS8 show partial overlapping. C) Comparison of the lesion profiles in M109MB, 301C and 301V show near complete overlapping, particularly between M109MB and 301C. Brain-scoring positions are medulla (1), cerebellum (2), superior colliculus (3), hypothalamus (4), thalamus (5), hippocampus (6), septum (7), retrosplenial and adjacent motor cortex (8), and cingulate and adjacent motor cortex (9). (TIF) [file ppat.1002370.s006.tif]

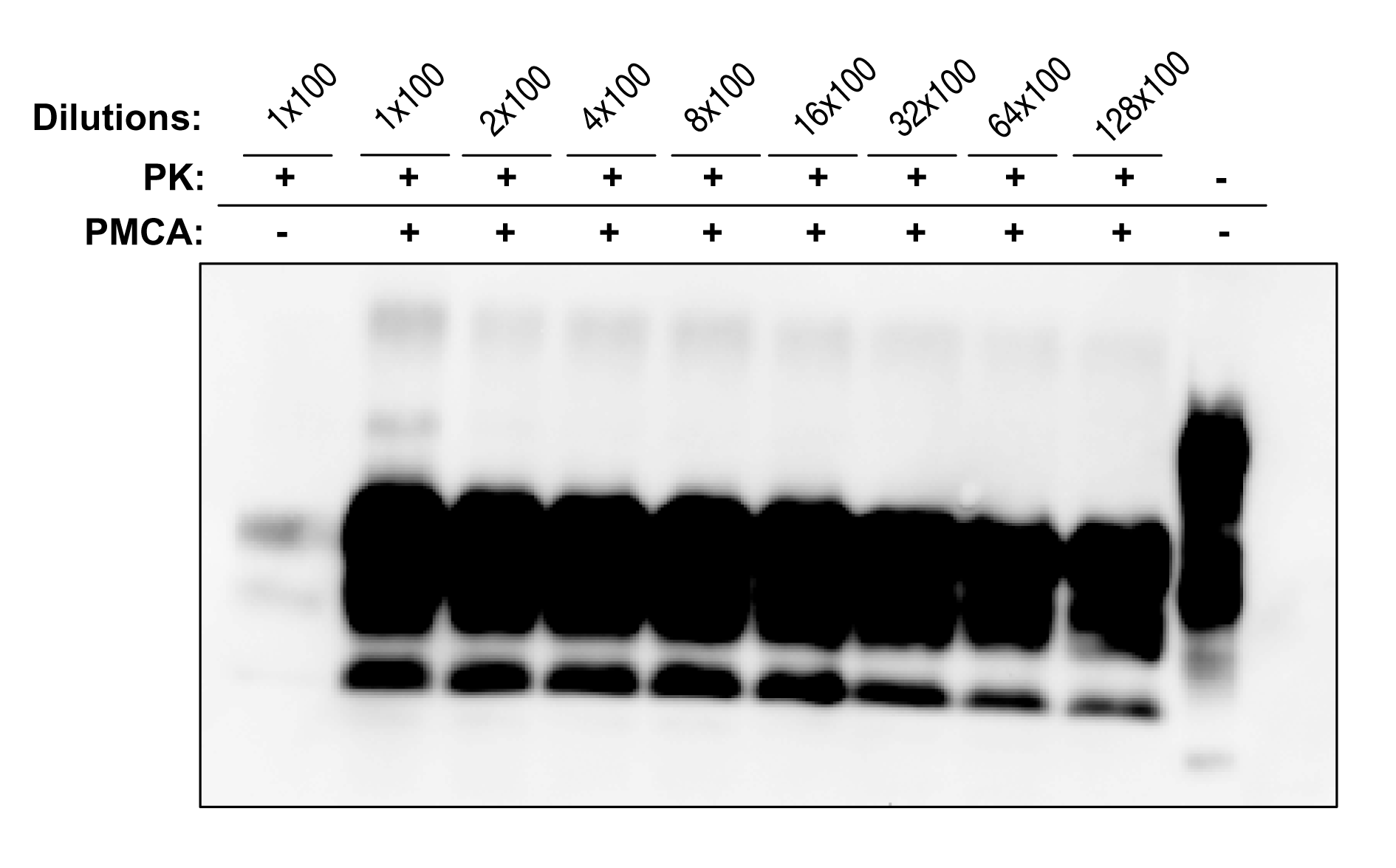

Supplement: Figure S7 — A test of the efficiency of PMCA. Two-fold serial dilution was prepared from 1∶100 to 1∶12800 using v586 inculum and vole M109M substrate. Samples were amplified for a single round of 24 hours (48 cycles of sonication/incubation). The first lane shows the unamplified “frozen” 1∶200 dilution and the second lane shows the dilution curve after PMCA. Western blots were probed with SAF84 primary antibody. (TIF) [file ppat.1002370.s007.tif]

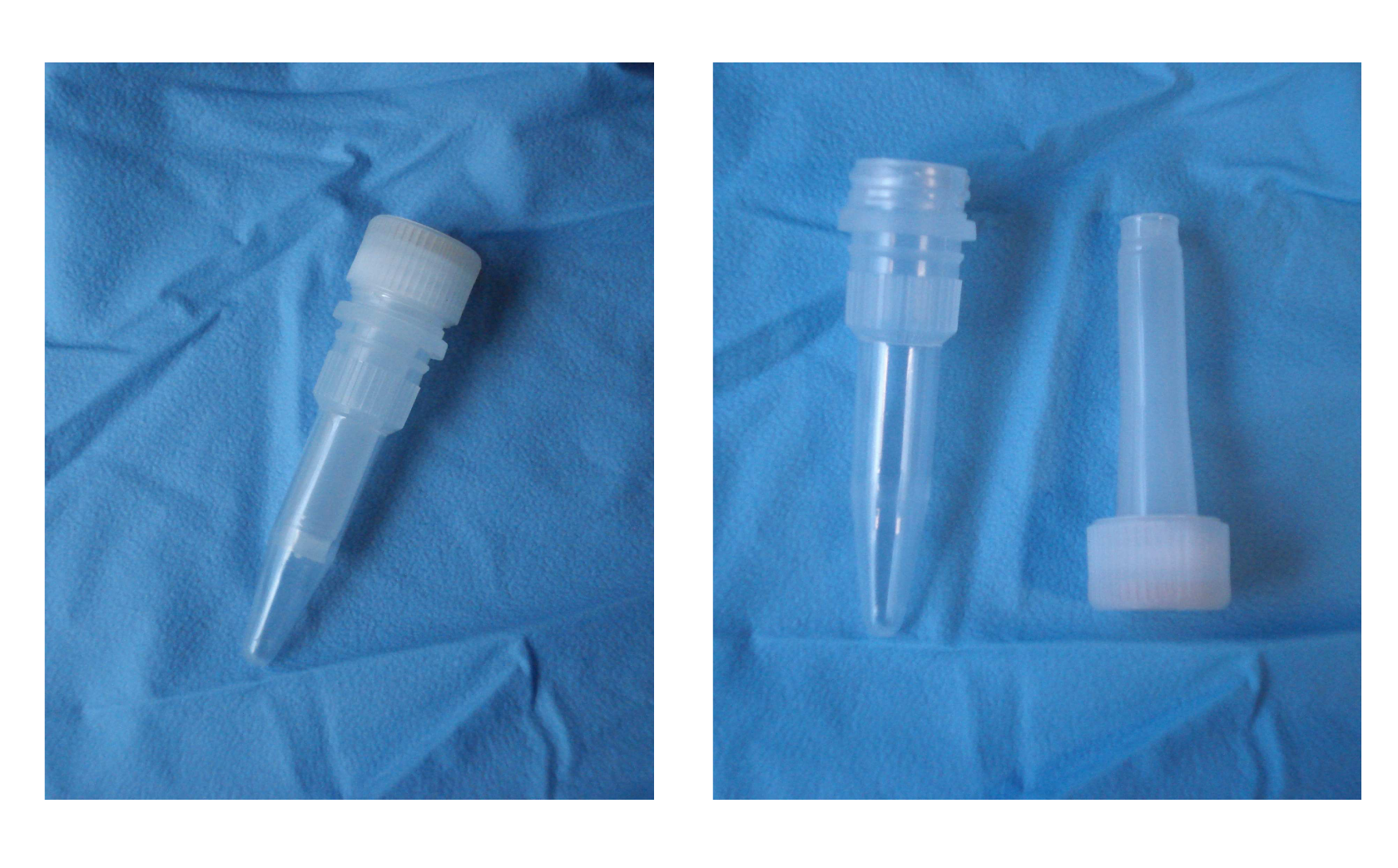

Supplement: Figure S8 — Screw-cap tube for PMCA. Containment of PrPSc during saPMCA was improved by using Sarstedt 0.5 mL Multiply-Safecup with 100 µl volume limitation. This tube is easy to handle, the volume limiter confines reaction mix to the bottom of the vial, where it is better exposed to the effect of the ultrasound, keeping the substrate away from the rim of the tube. Reaction sensitivity is comparable with 0,2 mL PCR tubes, while control of cross-contamination is much more efficient. (TIF) [file ppat.1002370.s008.tif]

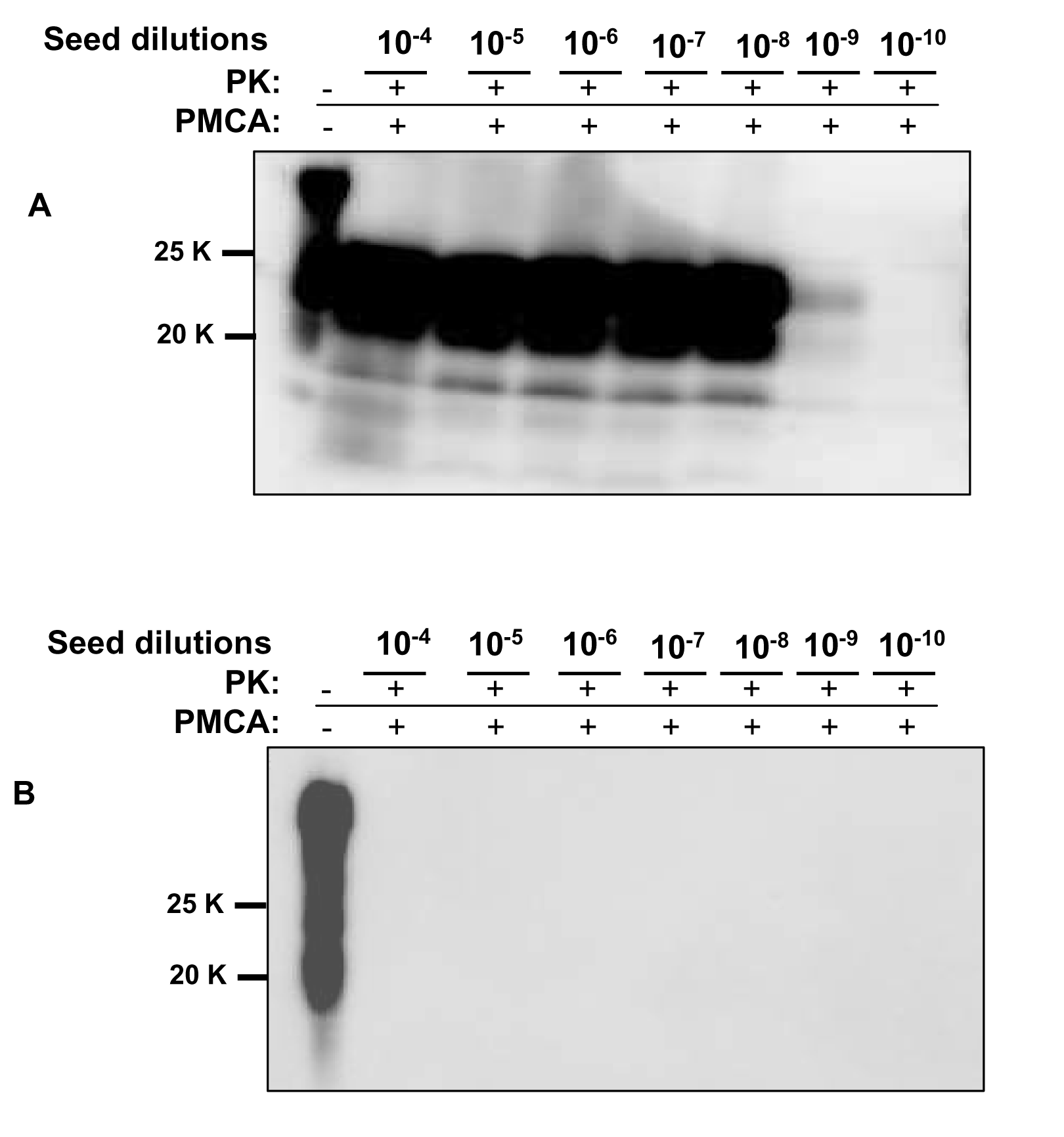

Supplement: Figure S9 — Evaluating the ability of substrate to amplify PrPSc after 48 h of PMCA. Logarithmic dilutions of v586 seed were prepared from 10−4 to 10−10 using either: freshly prepared M109M vole substrate (A) or a substrate carrying the same PrP genotype previously submitted to repeated cycles of incubation/sonication for 48 hours (B). Dilutions were submitted to a single round of 48 hours of PMCA. Western blots were probed with SAF84 primary antibody. (TIF) [file ppat.1002370.s009.tif]
