# Supplementary material for: Ultra-Efficient PrPSc Amplification Highlights Potentialities and Pitfalls of PMCA Technology
Source: PLoS Pathog. 2011 Nov 17;7(11):e1002370. doi: 10.1371/journal.ppat.1002370 (PMC3219717; doi:10.1371/journal.ppat.1002370)
Supplement: Table S1 — Supposedly de novo PrPSc appearance in unseeded PMCA reactions using bank vole substrates. (DOC) [file ppat.1002370.s010.doc]

**Table S1.** **Supposedly *de novo* PrP^Sc^ appearance in unseeded PMCA reactions using bank vole substrates**

| **N° exp** | **PrP genotype** | **Brain treatment** | **Hours per rounds** | **R1** | **R2** | **R3** | **R4** | **R5** | **Age (Y/M/O)** |
| --- | --- | --- | --- | --- | --- | --- | --- | --- | --- |
| 1 | M109M | Perfused | 48h | 0/4 | 0/4 | 0/4 | 3/4 | 3/4 | O |
| 2 | M109M | Perfused | 48h | 0/4 | 0/4 | 0/4 | 1/4 | 1/4 | O |
| 3 | M109M | Perfused | 48h | 0/4 | 0/4 | 0/4 | 1/4 | 1/4 | Y |
| 4 | M109M | Perfused | 48h | 0/4 | 0/4 | 1/4 | 1/4 | 1/4 | M |
| 5 | M109M | Perfused | 48h | 0/4 | 0/4 | 0/4 | 0/4 | 0/4 | Y |
| 6 | M109M | Perfused | 48h | 0/4 | 0/4 | 0/4 | 0/4 | 0/4 | Y |
| 7 | M109M | Perfused | 48h | 0/4 | 0/4 | 0/4 | 2/4 | 2/4 | Y |
| 8 | M109M | Perfused | 48h | 0/4 | 0/4 | 0/4 | 0/4 | 0/4 | M |
| 9 | M109M | Perfused | 48h | 0/4 | 0/4 | 0/4 | 0/4 | 2/4 | Y |
| 10 | M109M | Perfused | 48h | 0/6 | 2/6 | ND | ND | ND | Y |
| 11 | M109M | Perfused | 48h | 0/4 | 3/4 | ND | ND | ND | Y |
| 12 | M109M | Perfused | 48h | 0/4 | 2/4 | ND | ND | ND | Y |
| 13 | M109M | Perfused | 48h | 0/4 | 0/4 | 1/4 | 3/4 | ND | Y |
| 14 | M109M | Perfused | 48h | 0/4 | 1/4 | 2/4 | 4/4 | ND | Y |
| 15 | M109M | Perfused | 24h | 0/4 | 0/4 | 3/4 | 4/4 | 4/4 | Y |
| 16 | M109M | Perfused | 24h | 0/4 | 0/4 | 2/4 | 4/4 | 4/4 | Y |
| 17 | I109I | Perfused | 48h | 0/4 | 0/4 | 1/4 | 4/4 | 4/4 | O |
| 18 | I109I | Perfused | 48h | 0/4 | 0/4 | 0/4 | 0/4 | 0/4 | Y |
| 19 | I109I | Perfused | 48h | 0/12 | 0/12 | 3/12 | 4/12 | 5/12 | O |
| 20 | I109I | Perfused | 48h | 0/6 | 0/6 | 0/6 | 0/6 | 0/6 | Y |
| 21 | I109I | Perfused | 48h | 0/4 | 1/4 | 3/4 | 4/4 | 4/4 | O |
| 22 | I109I | Perfused | 48h | 0/4 | 0/4 | 1/4 | 1/4 | 3/4 | Y |
| 23 | I109I | Perfused | 48h | 0/4 | 3/4 | 4/4 | 4/4 | 4/4 | Y |
| 24 | M109M - Wild vole 1*  Wild vole | Not perfused | 48h | 0/6 | 0/6 | 0/6 | 1/6 | 1/6 | n.d. |
| 25 | M109M - Wild vole 2* | Not perfused | 48h | 0/6 | 0/6 | 0/6 | 1/6 | 1/6 | n.d. |

**Footnote to table S1 :** Two different vole genetic backgrounds were used in unseeded PMCA experiments. Both M109M and I109I vole *Prnp* genotypes are equally represented in the facility of the Istituto Superiore di Sanità. For these studies, we handled both genotypes in the same manner because we did not know how they were going to behave in relation to the generation of vole prions *de novo*. Twenty-five independent experiments were performed in quadruplicate, or in replicates of six, or twelve, using substrates from young (Y=less than 3 months) medium (M=between 3 and 6 months) and old (O=more than 6 months) animals.

Two wild bank voles were trapped in a rural area 40 miles from Rome, about 3 miles from Sutri in the province of Viterbo. This area is far from any plausible source of prions (hospitals, abattoirs, etc.). The animals were captured alive using new Sherman traps. They were immediately transported in an environment completely free of prions. The two animals carried M109M genotype and an additional PrP polymorphism, N43S. Voles from the ISS breeding colony were all S43S, while wild voles 1 and 2 were N43S and N43N, respectively.
