# Supplementary material for: Ultra-Efficient PrPSc Amplification Highlights Potentialities and Pitfalls of PMCA Technology
Source: PLoS Pathog. 2011 Nov 17;7(11):e1002370. doi: 10.1371/journal.ppat.1002370 (PMC3219717; doi:10.1371/journal.ppat.1002370)
Supplement: Table S2 — Transmission of putative de novo vole prion strains by intra-cerebral inoculation in voles. (DOC) [file ppat.1002370.s011.doc]

**Table S2 : Transmission of putative *de* *novo* vole prion strains by intra-cerebral inoculation in voles**

| **Inoculum** | **PrP genotype of recipient** | **I passage** | | | | | **II passage** |
| --- | --- | --- | --- | --- | --- | --- | --- |
|  |  | **Sympt. (+)** | **Pathol. (+)** | **PrPSc (+)** | **Survival time (days±SD)** | **Transmission rate** | **Survival time (days±SD)** |
| M109M Strain **A** | M109M | 12/12 | 12/12 | 12/12 | 138±5 | 100**%** | 124±3 |
| M109M Strain **B** | M109M | 13/13 | 13/13 | 13/13 | 84±4 | 100**%** | 72±4 |
| I109I Strain **A** | I109I | 10/10 | 10/10 | 10/10 | 216±13 | 100**%** | ND |
| I109I Strain **B** | I109I | 12/12 | 12/12 | 12/12 | 184±14 | 100**%** | ND |
| M109M NBH | M109M | 0/10 | 0/10 | 0/10 | >600 | 0**%** | ND |
| I109I NBH | I109I | 0/10 | 0/10 | 0/10 | >600 | 0**%** | ND |

**Footnote to table S2**: Putative *de novo* A and B strains generated in unseeded M109M or I109I saPMCA reactions were characterized by inoculation in the respective genetic lines of voles, along with appropriate negative controls (normal brain homogenates, NBH). While voles infected with NBH are healthy after >600 days post-inoculation, all inocula containing PrP^res^ transmitted a TSE in voles (see also Figures S4 and S5). The survival times were different among the different strains, with B strains showing shorter survival times than A strains. We performed second passages into M109M voles, which showed a slight shortening of the survival times and preservation of the phenotypic features observed after primary passage (data not shown).
