# Supplementary material for: Ultra-Efficient PrPSc Amplification Highlights Potentialities and Pitfalls of PMCA Technology
Source: PLoS Pathog. 2011 Nov 17;7(11):e1002370. doi: 10.1371/journal.ppat.1002370 (PMC3219717; doi:10.1371/journal.ppat.1002370)
Supplement: Table S3 — Comparison of seeded and unseeded saPMCA products by intra-cerebral inoculation in voles. (DOC) [file ppat.1002370.s012.doc]

**Table S3. Comparison of seeded and unseeded saPMCA products by intra-cerebral inoculation in voles**

| **PMCA-passaged strains** | **PrP^res^ types** | **Transmission in vivo** | |
| --- | --- | --- | --- |
|  |  | **Survival time (days±SD)** | **Transmission rate** |
| M109M Strain A | scrapie-like | 124±3 | 100% |
| vole – adapted SS8 | scrapie-like | 113±13 | 100% |
| M109M Strain B | BSE-like | 72±4 | 100% |
| vole –adapted 301C | BSE-like | 90±5 | 100% |
| vole – adapted 301V | BSE-like | 75±10 | 100% |

**Footnote to table S3:** All strains that were reproduced in vitro by 15 consecutive rounds of saPMCA using M109M vole substrate (see Figure S3) were infectious when inoculated intracerebrally in M109M voles, showing survival times similar to the *in vivo* passaged counterparts (data not shown). Among these, vole-adapted SS8, derived from ARQ/ARQ sheep [31] and characterized by a scrapie-like PrP^res^ pattern (Figure S3), and vole-adapted 301C and 301V, derived from mouse strains and characterized by a BSE-like PrP^res^ pattern (Figure S3), gave survival times similar to strains A and B, respectively. Furthermore, after *in vivo* passage in voles, strain A and SS8 showed similar PrP^res^ patterns (data not shown) and lesion profiles (Figure S6). Similarly, strain B, 301C and 301V gave identical PrP^res^ patterns (data not shown) and lesion profiles (Figure S6).
