# Supplementary material for: Ultra-Efficient PrPSc Amplification Highlights Potentialities and Pitfalls of PMCA Technology
Source: PLoS Pathog. 2011 Nov 17;7(11):e1002370. doi: 10.1371/journal.ppat.1002370 (PMC3219717; doi:10.1371/journal.ppat.1002370)
Supplement: Text S1 — Supporting results. Early experimental evidences indicated that the appearance of PrPSc in unseeded substrate samples was frequent after a variable number of saPMCA rounds. This finding was repeatedly observed in several different independent experiments and using numerous vole substrates (Table S1). PrPSc isolates from unseeded experiments were studied by western blot. Based on the differential electrophoretic mobilities, two distinct PrPSc types (named A and B) were recovered from M109M and I109I vole genotypes. Putatively de novo strains A and B were further passaged for up to 10 serial round of PMCA maintaining their distinctive electrophoretic mobility. (Figure S1). The PrPSc types, that were supposed to be of spontaneous origin, have been intensively studied using methods available for TSEs strain typing (Figures S2, S3, S4, S5, S6 and Tables S2 and S3). (DOC) [file ppat.1002370.s013.doc]

**Supporting results**

Early experimental evidences indicated that the appearance of PrP^Sc^ in unseeded substrate samples was frequent after a variable number of saPMCA rounds. This finding was repeatedly observed in several different independent experiments and using numerous vole substrates (Table S1).

Being PMCA carried out by skilled persons, taking maximum care to avoid all possible sources of contamination from the laboratory environment, the spontaneous appearance of PrP^Sc^ in tubes containing unseeded substrate, this data suggested that *de novo* generation was probably taking place into the reaction tubes. The retrospective analysis of data lead to consider positive control source of cross contamination into the sonicator. Indeed when unseeded samples were amplified in absence of any positive into the sonicator, we did not observe the appearance of spurious PrP^Sc^.

PrP^Sc^ isolates from 23 different unseeded experiments of vole saPMCA (see Table S1) were studied by western blot. Based on the differential electrophoretic mobilities, two distinct PrP^Sc^ types (named A and B) were recovered from M109M and I109I vole genotypes. Putatively *de novo* strains A and B were further passaged for up to 10 serial round of PMCA maintaining their distinctive electrophoretic mobility. (Figure S1).

The PrP^Sc^ types, that were supposed to be of spontaneous origin, have been intensively studied using methods available for TSEs strain typing and compared with well known TSEs strains in order to observe if spontaneous prions could be distinguished unequivocally from other vole TSEs strains (Figures S2, S3, S4, S5, S6 and Tables S2 and S3).
