# Supplementary material for: Ultra-Efficient PrPSc Amplification Highlights Potentialities and Pitfalls of PMCA Technology
Source: PLoS Pathog. 2011 Nov 17;7(11):e1002370. doi: 10.1371/journal.ppat.1002370 (PMC3219717; doi:10.1371/journal.ppat.1002370)
Supplement: Text S2 — Method section for supporting Figures and Tables. (DOC) [file ppat.1002370.s014.doc]

**Animals**

Bank voles (Myodes glareolus, formely Clethrionomys glareolus were obtained from the M109M and I109I breeding colonies at the Istituto Superiore di Sanità, Rome, Italy. Subjects were individually identified by passive integrated transponders, inoculated when weanlings (40–60 days) and kept in groups of two-four individuals per cage.

#### Inoculations and clinical follow-up

Animals were anaesthetized with ketamine and inoculated intracerebrally (i.c.) into the left hemisphere with 20 µl brain homogenate. Beginning one month after inoculation, animals were examined twice per week until the appearance of clinical symptoms, and then examined daily. We measured the survival time instead of the incubation time because of the differences among strains in the clinical phenotype of the disease. Diseased animals were sacrificed with carbon dioxide at the terminal stage of disease but before neurological impairment was such as to compromise welfare and, especially, adequate drinking and feeding. Survival time was calculated as the interval between inoculation and sacrifice or death.

#### Histopathology, immunohistochemistry and Western-blot analysis

After collection at sacrifice, each brain was cut parasagitally into two parts. The smaller one was stored at −80°C for biochemical studies. The other part was fixed in formalin for histology assessment (lesion profile) and immunohistochemistry analysis as described previously [28] . PK-resistant PrP was examined by Western blotting in SDS-PAGE gels and by PET blot, as previously described [28].

The monoclonal antibodies used in western blots, their epitopes and the working dilutions were as follow: SAF84, PrP residues 160–170, 1.2 µg/ml; 12B2, PrP residues 89–93, 2.4 µg/ml. Horseradish peroxidase-conjugated anti-mouse immunoglobulin (Pierce Biotechnology, Rockford, Illinois, United States) was used as secondary antibody (1:13000). The membranes were developed with an enhanced chemiluminescence method (SuperSignal Femto, Pierce). Chemiluminescence signal was detected with the VersaDoc imaging system (Bio-Rad) and was quantified by QuantityOne software (Bio-Rad).

Deglycosylation was performed by adding 18 µl of 0.2 M sodium phosphate buffer (pH 7.4) containing 0.8% Nonidet P40 (Roche) and 2 µl (80 U/ml) di N-Glycosidase F (Roche) to 5 µl of denaturated samples and by incubating overnight at 37°C with gentle shaking. Samples were then analysed by Western blotting as described above.
